# Bidentate Metal-Binding Motifs in US FDA Approved Small Molecule Drugs

**DOI:** 10.1021/acs.jmedchem.6c01319

**Published:** 2026-07-21

**Authors:** Jon T. Njardarson, Elisa Tomat

**Affiliations:** † Department of Chemistry and Biochemistry, 8041 University of Arizona, Tucson, Arizona 85721, United States; ‡ Institute of Inorganic Chemistry, Faculty of Chemistry, 27258 University of Vienna, 1090 Wien, Austria

## Abstract

The coordination of metal cations is integral to the mechanism of action of metal-binding pharmaceuticals such as scavengers for chelation therapy and metalloprotein inhibitors. Furthermore, clinically relevant interactions with endogenous metals were initially unrecognized for some drug classes and are still debated in some cases. In this Perspective, we survey the small molecules approved as drugs by the United States Food and Drug Administration (US FDA) looking for structural motifs wherein metal ions could potentially coordinate in a bidentate fashion. We focus on the formation of five- or six-membered chelate rings upon coordination with nitrogen, oxygen and sulfur donors. In addition to well-known metal-binding motifs such as hydroxamic acids, catechols and salicylic acids, our review highlights numerous drugs containing 1,3-dicarbonyl motifs and related oxygen donors. Nitrogen heterocycles are also featured prominently in this survey of bidentate motifs and a clear growth category in recent years.

## Graphical abstract

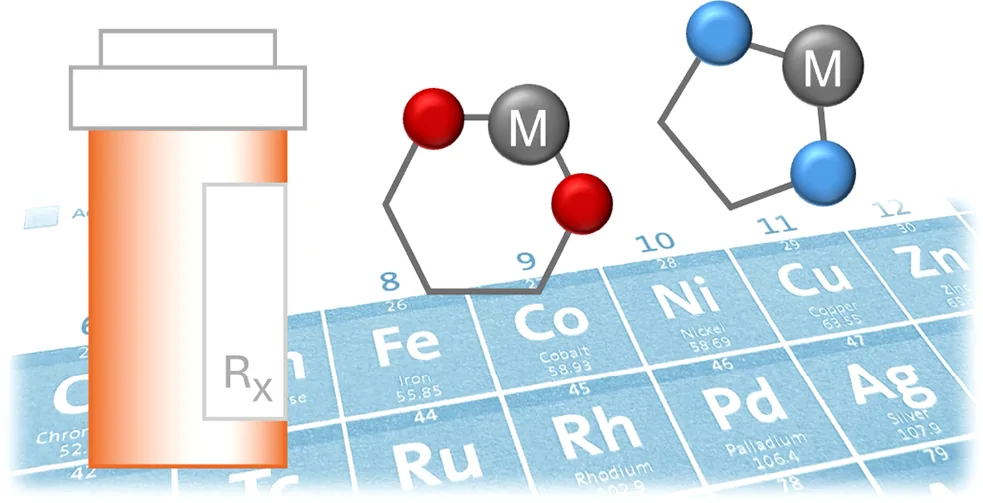

## Significance

The collection of US FDA approved small-molecule drugs presents a broad variety of structural motifs that are potentially suitable for bidentate metal coordination. This structural analysis highlights a range of cases: from those involving documented metal interactions of clinical relevance to those for which metal binding has not been reported to date. Considerations of coordination chemistry could assist the design of new drugs as well as mechanistic investigations of existing therapeutics.

## Introduction

Multiple historical documents, including the Ebers Papyrus dated ca. 1550 BCE, attest to the use of metals to treat diseases since antiquity. Metal-containing compounds have certainly played pivotal roles in the history of pharmacology. For instance, arsenic-based Salvarsan, discovered in 1909 by Paul Erlich and used widely for the treatment of syphilis, was one of the most prescribed drugs of the early 20th century. The discovery of cisplatin as an anticancer agent in the 1970s then cemented the position of medicinal inorganic chemistry in modern medicine.^1,2^ Today, metal complexes are employed clinically for a broad variety of properties both in diagnosis and treatment.^3–5^ Among examples of metal-based therapeutics (Figure 1), cisplatin and auranofin interact directly with biomolecules via covalent binding, whereas radioactive lutetium-177 complexes damage tumor tissue through the emission of beta particles in targeted anticancer radiotherapy. In diagnostic applications (Figure 2), β-emitting gallium-68 compounds enable positron emission tomography (PET), and gadolinium complexes enhance proton relaxation for magnetic resonance imaging (MRI).

**Figure 1:**
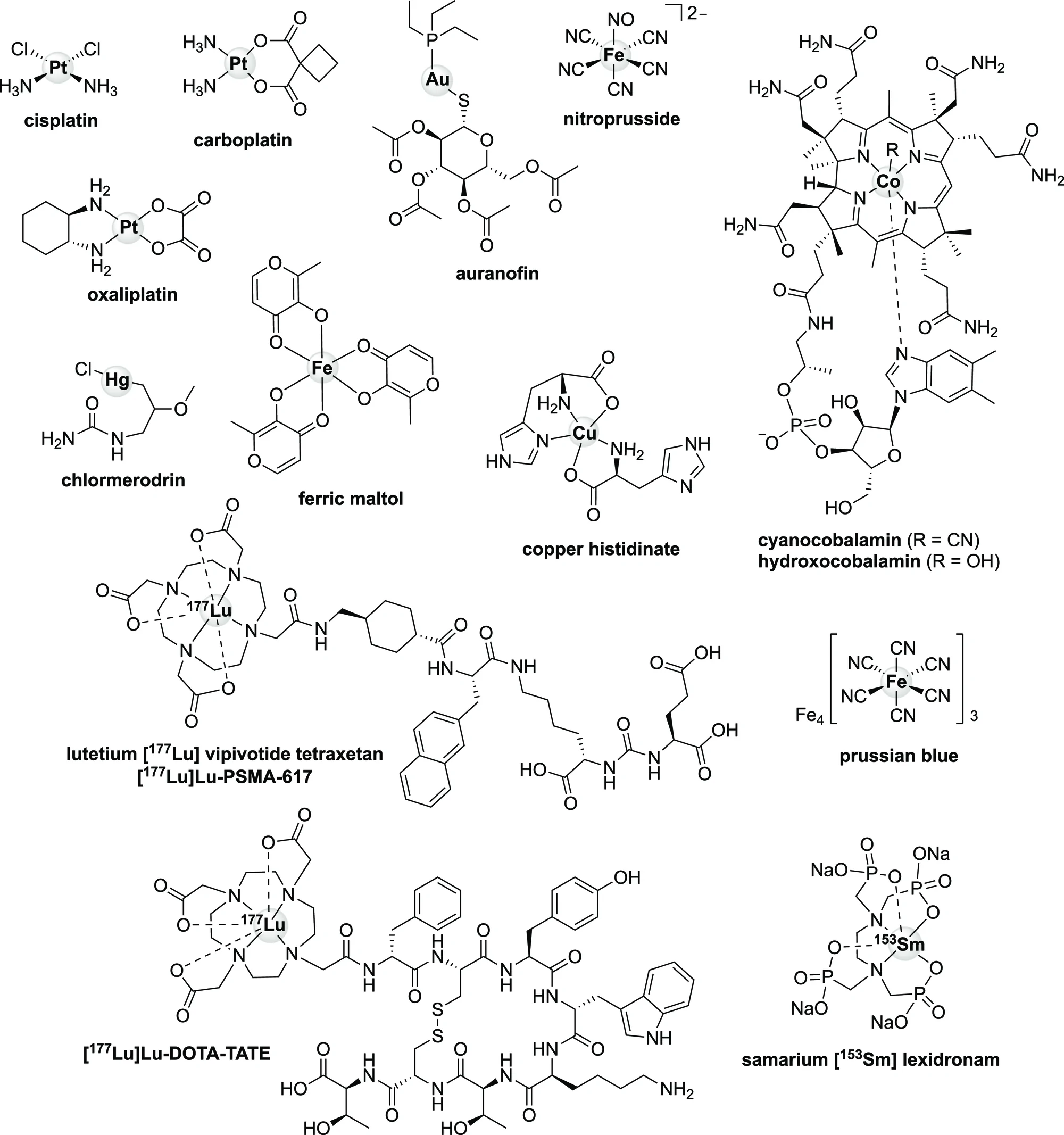
Examples of US FDA approved metal complexes for therapy.

**Figure 2:**
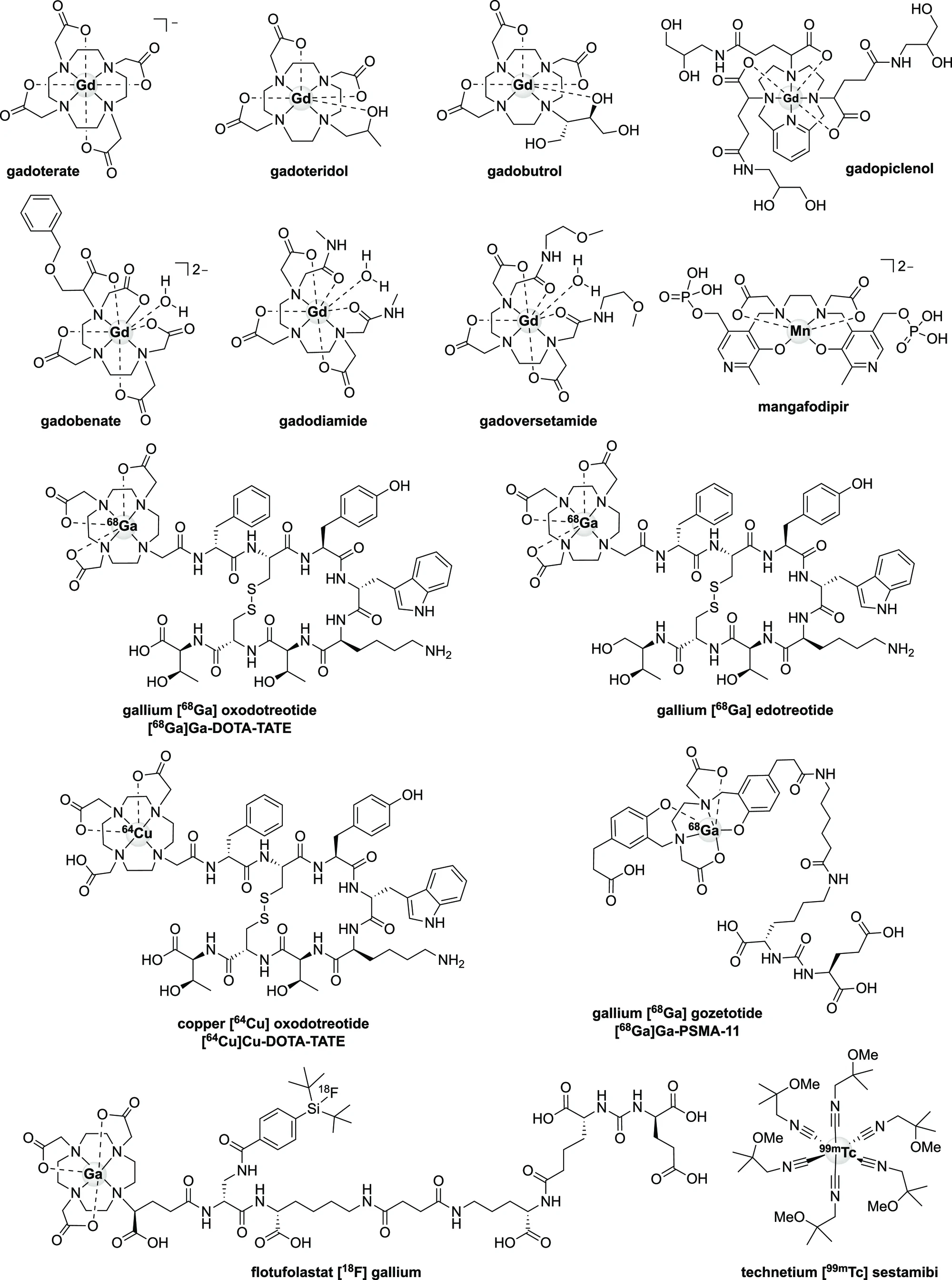
Examples of US FDA approved metal complexes for diagnostics.

Many simple metal salts included in dietary supplements are not featured in our summary figures because they are not regulated as drugs; however, supplementation with certain transition metal compounds requires careful monitoring and has received FDA approval: for instance, ferric maltolate for iron deficiency and cyanocobalamine for vitamin B12 deficiency (Figure 1). Copper histidinate (shown in the coordination mode crystallized at physiological pH in Figure 1)^6^ was approved this year as the first treatment for Menkes disease in pediatric patients. Given our focus on metal complexes, some FDA approved inorganic drugs (e.g., antineoplastic arsenic trioxide and mood stabilizer lithium carbonate) were also left out of our summary figures.

New approaches in medicinal inorganic chemistry are in various stages of experimental, preclinical, and clinical development. Several metal complexes, including organometallic compounds, photoactivated metallopharmaceuticals, and metal-based inhibitors, are being pursued in anticancer applications.^7–10^ The immune response associated with metal-based drugs is emerging as an important area of investigation.^11^ Screening campaigns of compounds sourced through the bioinorganic community are highlighting the untapped potential of metal complexes in antimicrobial drug discovery.^12^ In this context, the high chemical diversity of metal complexes, particularly their three-dimensional shapes, is poised to enhance the reach of libraries for fragment-based drug discovery.^13^ Furthermore, the one-electron redox chemistry of several transition metals and the interactions of metal complexes with reactive oxygen and nitrogen species can be exploited in therapeutic agents that impact the intracellular redox homeostasis.^14^

Whereas metal-containing pharmaceuticals include metals in their formulation, metal-binding pharmaceuticals engage with metal ions in the organism following administration orally or intravenously. The classic metal-binding pharmaceuticals, typically described as chelators or scavengers, coordinate metals with high affinity to form complexes that are rapidly excreted. These pharmaceuticals constitute the standard of care for metal overload disorders. For example, hexadentate deferoxamine, bidentate deferiprone and tridentate deferasirox (Figure 3) are employed as chelators to treat iron overload associated with hemochromatosis and thalassemia.^15,16^ Dithiolates like meso-2,3-dimercaptosuccinic acid and D,L-2,3-dimercapto-1-propanesulfonic acid (Figure 3) are important tools for the treatment of acute and chronic intoxications due to lead and mercury exposure.^17^ Ethylenediaminetetraacetic acid (EDTA) and its sodium and calcium salts are also used primarily in the context of exposure to toxic metals.^18^ Furthermore, d-penicillamine and trientine are employed as copper chelators for the treatment of Wilson’s disease, an inherited disorder characterized by excess copper accumulation.^19^

**Figure 3:**
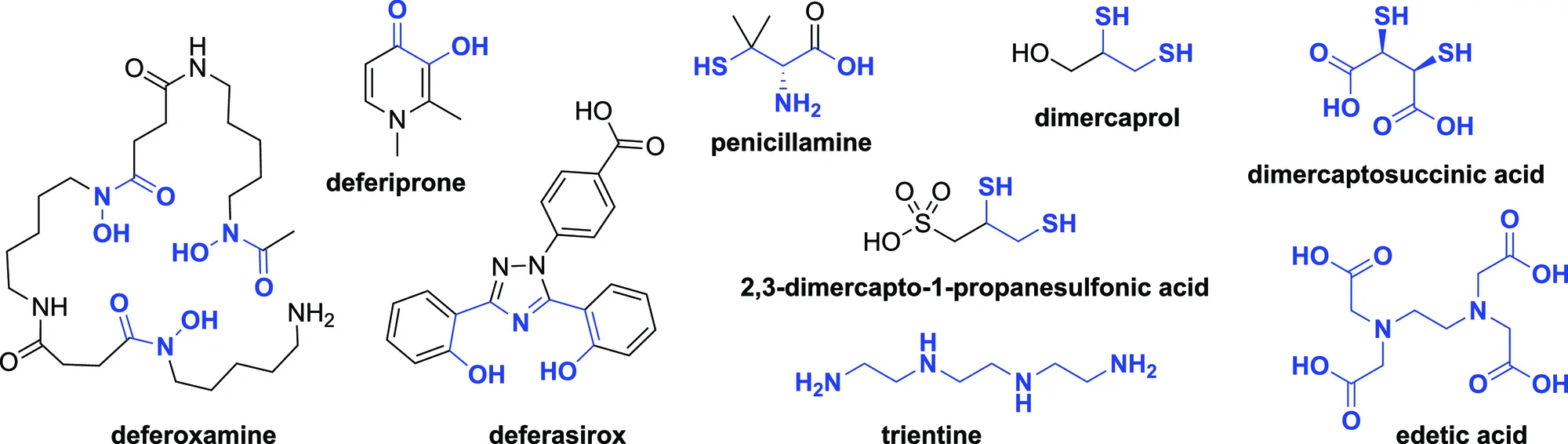
Examples of metal-binding pharmaceuticals used as chelators of excess metal ions.

Interactions between administered drugs and metal ions in the organism are not exclusive to chelators and scavengers for the treatment of metal overload. Metalloenzyme inhibitors, including several FDA approved drugs (vide infra), are specifically designed to interact with the metal center(s) at the active site of the enzyme. These small molecules recognize a binding pocket within the metalloenzyme while also engaging the metal center in the active site either through a single donor group or through multidentate binding unit (i.e., metal-binding pharmacophore).^20,21^ In the pipeline, small molecules that alter the availability and/or distribution of transition metals (e.g., chelators, prochelators, ionophores) are currently under investigation for the treatment of several conditions, including neurodegenerative disorders and cancer.^22–24^

Unwanted metal binding is also a well-recognized issue in pharmacology. For instance, the efficacy of several drugs is affected by coadministration with metal supplements, particularly iron.^25^ In drug discovery, metal binding is a component of the chemistry that has led to the classification of certain compounds as PAINS, i.e., Pan-Assay Interference Compounds, which tend to register as hits in multiple screens and then cannot be further optimized.^26^ For instance, metal binding can produce false positives in multiple assays by affecting the function of a protein or the chemistry of a reagent. In addition, compounds that have high affinity for metal cations are susceptible to metal contamination, which also impacts screening results.^27^

Between the targeted metal binding of scavengers and metalloenzyme inhibitors and the unwanted metal binding in screening campaigns and drug–supplement interactions, coordination of endogenous metals also appears in drug discovery as part of the primary mechanism of action or as a component of a polypharmacological drug profile. For instance, antibiotic natural products of the bleomycin class (vide infra) are activated through metal coordination as they form redox-active iron and copper complexes that ultimately lead to DNA damage.^28^ Conversely, metal interactions are generally included in the factors contributing to the complex polypharmacology of salicylate drugs (vide infra).

With these considerations in mind, and based solely on a structural analysis, we sought to identify potential bidentate metal-binding motifs within the structures of US FDA approved small molecule drugs. We focused on structural motifs featuring common metal-binding groups (e.g., carboxylate, phenol, imine, aromatic nitrogen heterocycle, thiol) such that metal coordination would result in a 5- or 6-membered chelate ring. In other words, we selected for motifs that would benefit from the chelate effect—the increased stability of complexes of bidentate ligands (relative to that of complexes with monodentate analogs) particularly when forming optimal ligand–metal–ligand bite angles (i.e., 5- or 6-membered metallacycles). For instance, all the metal scavengers used in medicinal settings (Figure 3) are obvious hits for this search.

The ability of a certain small molecule to engage endogenous metals will naturally depend on its respective binding affinities and on specific metal concentrations, which are dynamic and diverse throughout different biological environments (e.g., blood plasma, cytosol, various intracellular compartments). Sodium, potassium and magnesium ions are abundant (i.e., millimolar intracellularly) and engage Lewis bases in interactions that are primarily electrostatic and kinetically labile.^29^ Conversely, chelatable pools of essential transition metals are tightly regulated in the cytoplasm at low or very low concentrations (i.e., micromolar for iron, picomolar for zinc and even lower for copper)^30–32^ and have the potential to form thermodynamically and kinetically stable complexes featuring covalent bonds with multidentate ligands. Because of the sophisticated roles of transition metals in biology and human physiology, compounds that affect their homeostasis and/or redox chemistry have the potential to elicit medicinally relevant effects and may offer therapeutic opportunities.^33^

The results of our survey of bidentate motifs are shown in the gallery of figures presented in this Perspective: each figure highlights a specific group of structural motifs (shown in blue) through relevant examples and includes the international nonproprietary name (INN)^34^ of each drug. Compounds featuring more than one bidentate motif appear in more than one figure. In some figures, the structures of three or more drugs of the same class are grouped in a dashed-line box. Peptidic chains were not included in our survey and, although our figures include over three hundred structures, it is possible that this visual analysis overlooked some examples. Furthermore, the list of FDA approved drugs is continuously changing, and some of the compound included here may have been withdrawn after the original approval or may be withdrawn in the future.

## DISCUSSION

Our structural analysis gathered drugs with well-documented metal-binding properties together with drugs that are not designed or expected to coordinate metals. Notably, our survey does not take into consideration several factors that could make metal-binding units largely ineffective, such as protonation equilibria, conformational distribution, and steric bulk. Even when metal coordination is demonstrated in the chemistry laboratory, the resulting metal complex may be of limited interest for medicinal applications. In addition, the presence in a small-molecule drug of a certain bidentate structural motif does not imply necessarily that coordination of endogenous metals occurs and is physiologically relevant. In some cases, however, metal interactions have been associated with observable effects, and our commentary includes a selection of reported examples, particularly those potentially relevant to the clinical profiles of the compounds. Several common bidentate motifs emerge from our collection of summary figures, which may provide new clues on potential interactions of clinically approved drugs with metal cations in the organism.

### Bidentate Motifs Featuring Oxygen Donors

The bidentate hydroxamate motif is the most widely studied metal-binding pharmacophore in drug discovery efforts targeting metalloenzymes. Although numerous campaigns using hydroxamates to target matrix metalloproteinases did not ultimately receive clinical approval, several hydroxamates were identified in the 1990s as potent inhibitors of histone deacetylases (HDACs) and therefore potential anticancer drugs. These efforts led to the development of FDA-approved HDAC inhibitors vorinostat, panobinostat, and belinostat (Figure 4), which employ the hydroxamate moiety to engage the Zn­(II) center at the catalytic site of the targeted enzymes.^35,36^ An additional hydroxamate-based HDAC inhibitor, givinostat (Figure 4), was approved more recently for the treatment of Duchenne muscular dystrophy.^37^ Hydroxyurea and acetohydroxamic acid (Figure 4) also feature primary hydroxamate motifs (i.e., with unsubstituted hydroxylamines) and are employed primarily for the treatment of sickle cell anemia and urinary tract infections, respectively. The complex coordination and redox chemistry of hydroxyurea and primary hydroxamates, particularly with respect to endogenous iron,^38^ likely contributes to their composite pharmacological profiles. For instance, hydroxamate-based HDAC inhibitors were found to impact iron homeostasis and ROS production,^39,40^ but also to display iron-dependent (and HDAC-independent) neuroprotective effects.^41^

**Figure 4:**
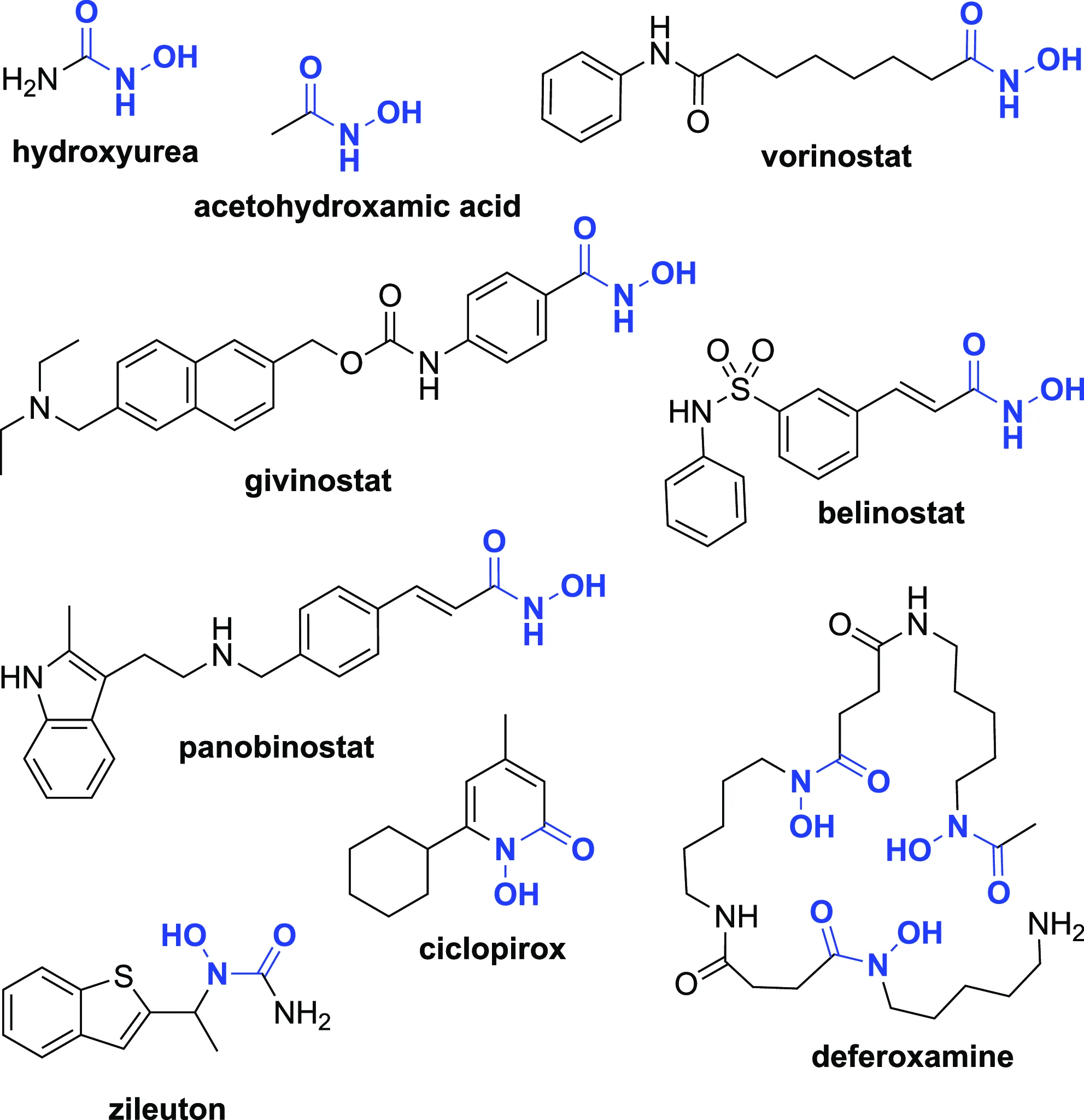
Hydroxamate- and hydroxyurea-containing motifs in metalloprotein inhibitors and other metal-binding drugs.

Substituted hydroxamates are found in several other metal-binding drugs. The 5-lypoxygenase inhibitor zileuton (Figure 4) is thought to interact with the iron center of the enzyme and is approved for the treatment of asthma.^42,43^ *N*-hydroxypyridone ciclopirox (Figure 4) is a common antifungal in topical preparations and its iron-binding properties have been related to the observed antifungal^44^ and antibacterial^45^ properties through multiple mechanisms of action. Finally, deferoxamine (Figures 3 and 4), which features three hydroxamate moieties, is a well-established hexadentate chelator employed for the treatment of iron overload (vide supra).

The most frequently appearing bidentate metal-binding motifs in our survey are 1,3-dicarbonyl chelates and their derivatives (Figures 5 and 8), which are suitable to coordinate metal cations as monoanionic ligands forming six-membered rings with two oxygen donors bound to the metal center.

**Figure 5:**
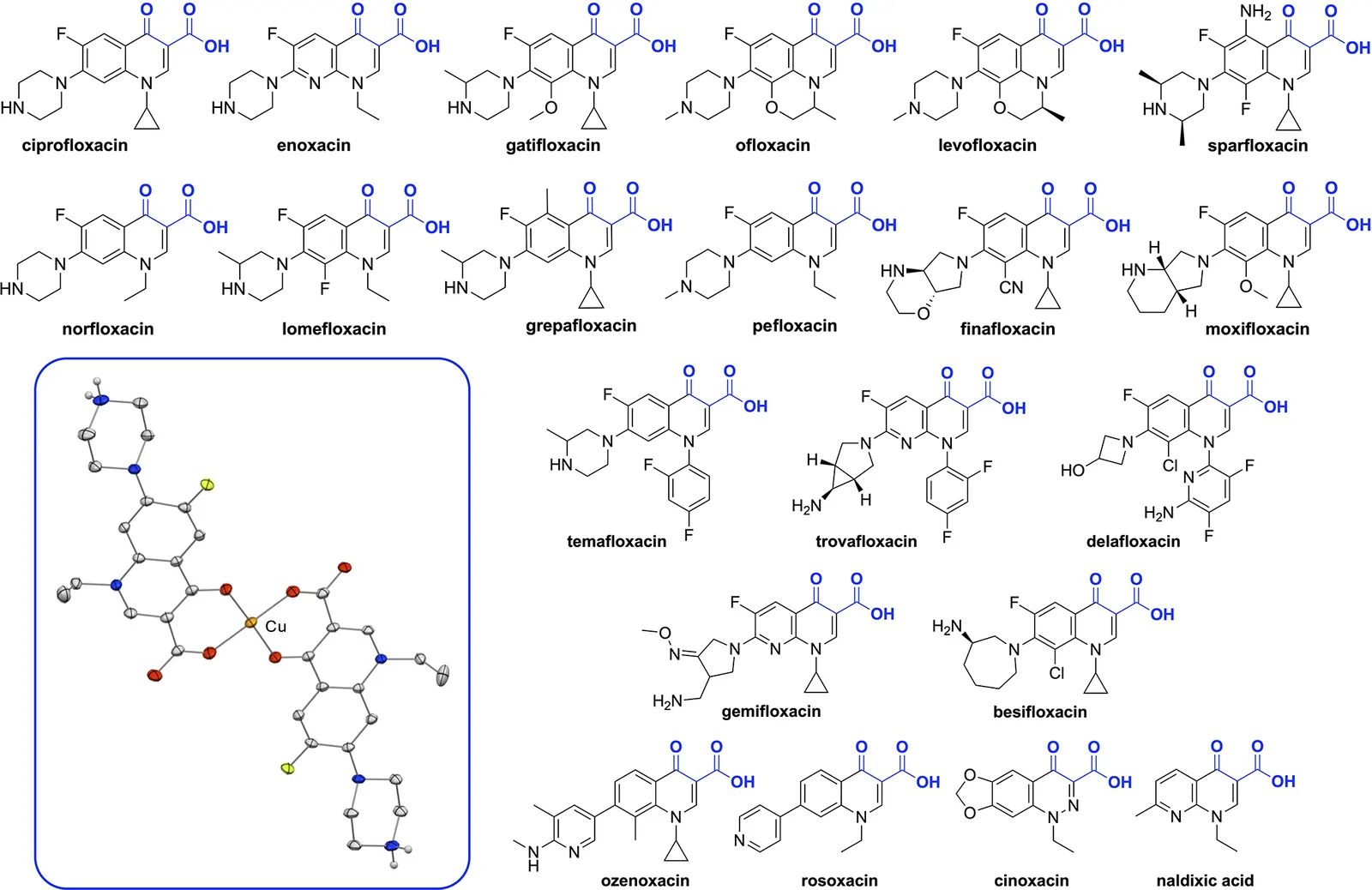
Floxacin family of antibiotic drugs. Inset: crystal structure of a norfloxacin Cu­(II) complex featuring bidentate coordination of the 4-quinolone-3-carboxylate motif.^53^ Two chloride counterions, six crystallization water molecules, and all carbon-bound hydrogen atoms are not shown. CCDC: 871610.

**Figure 6:**
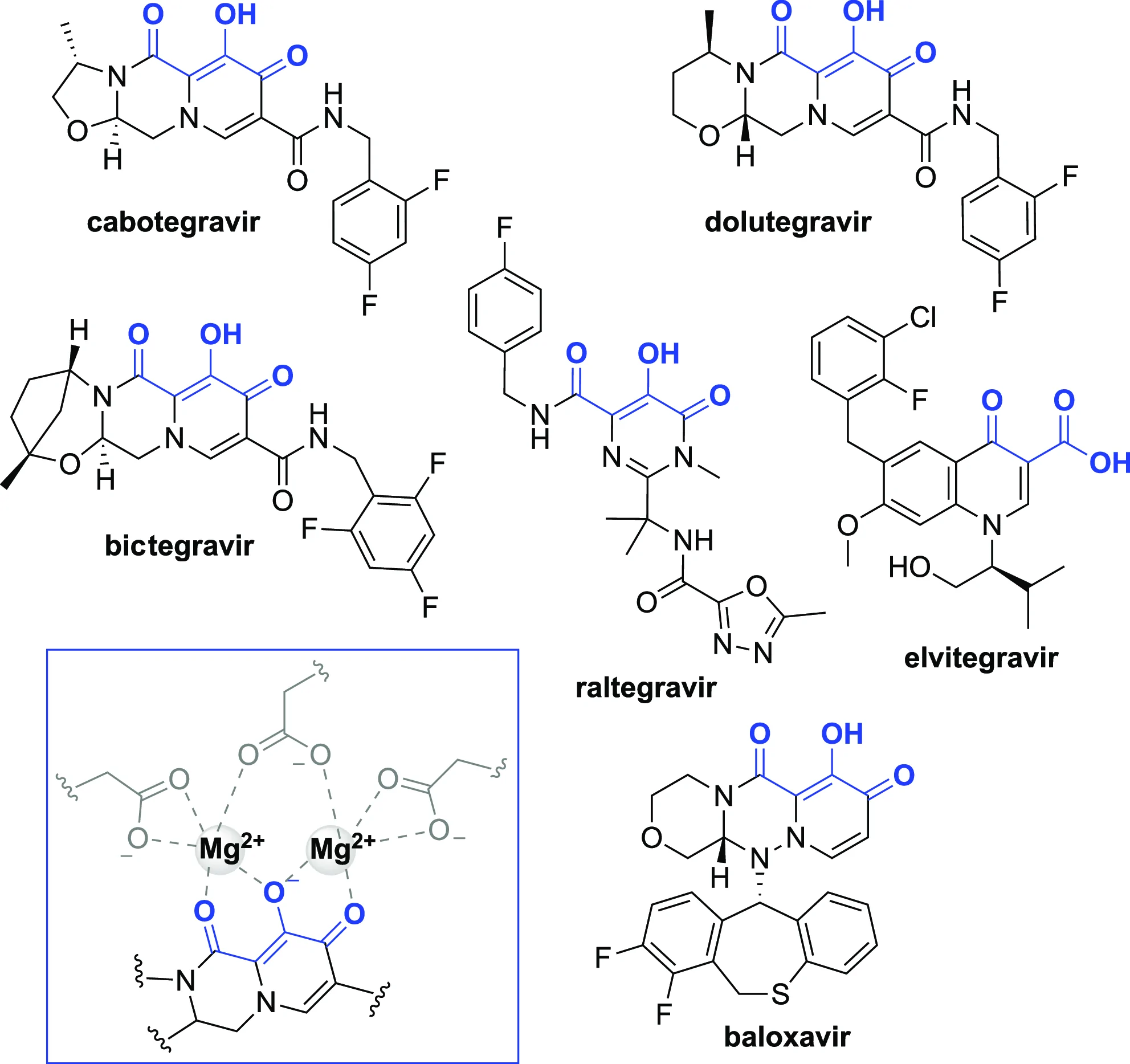
Antiviral drugs featuring bis-bidentate motifs with three oxygen donors. Inset: simplified scheme of the magnesium-mediated interaction of “gravir” drugs with three carboxylate residues at the active site of HIV-1 integrases.

**Figure 7:**
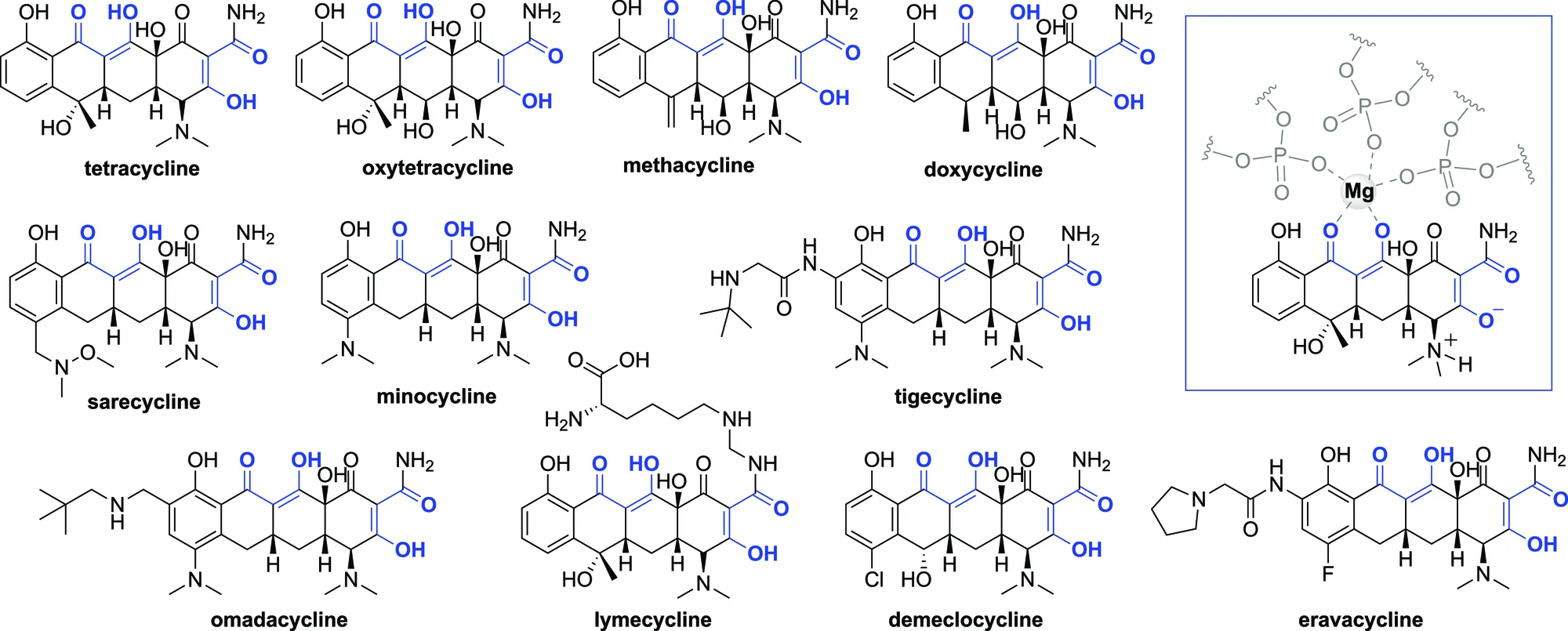
Tetracycline family of antibiotic drugs. Inset: simplified scheme of the interactions of the tetracycline magnesium complex with the phosphate groups of the S30 ribosomal subunit.

**Figure 8:**
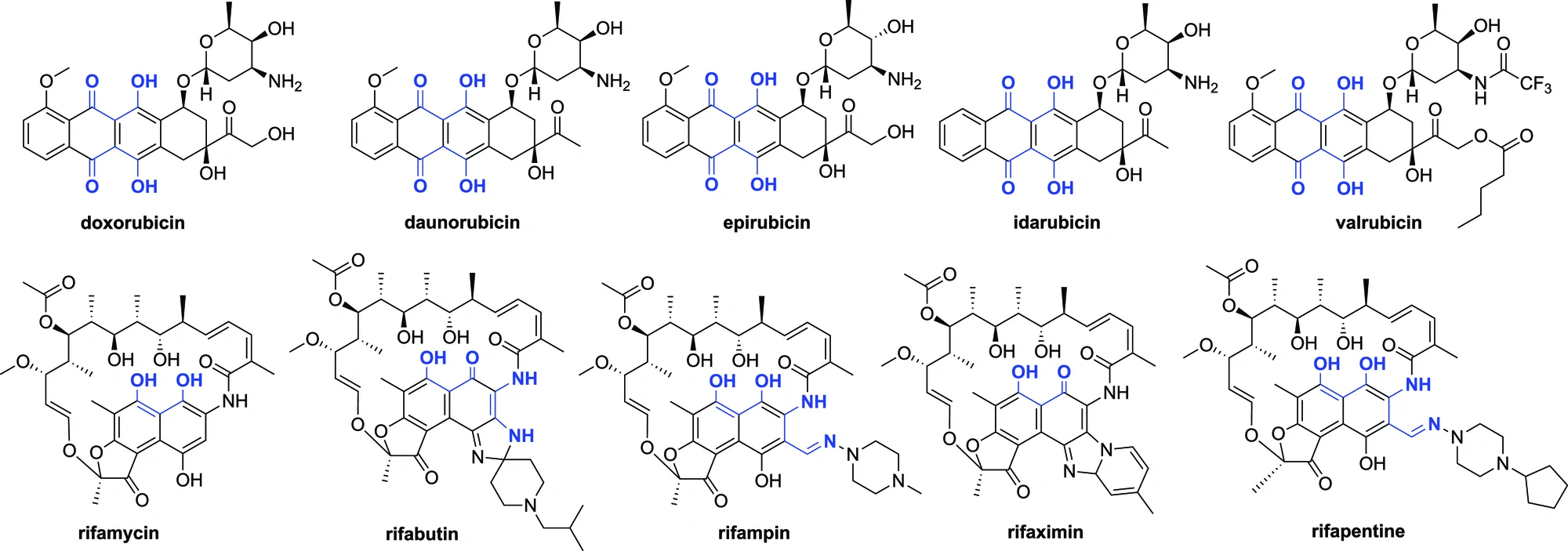
Anthracycline and rifamycin families of small molecule drugs.

Members of the floxacin family of drugs (Figure 5) are broad-spectrum antibiotics targeting DNA gyrase and/or topoisomerase IV in Gram-positive and Gram-negative bacteria.^46,47^ They share a characteristic 4-quinolone-3-carboxylic acid motif, and the bidentate coordination of magnesium mediates their interactions with the target DNA-enzyme complex, which was captured in multiple crystal structures.^48,49^ The 4-quinolone-3-carboxylate was also employed in the formation of several metal complexes of floxacin drugs with divalent and trivalent metal ions.^50–52^ The typical binding mode through the 4-quinolone-3-carboxylate motif is illustrated by one of the reported Cu­(II) complexes (Figure 5).^53^ The reported biological activities of the complexes include in some cases enhanced antimicrobial effects, as well as new antifungal, antiparasitic, and anticancer activities. Importantly, metal coordination of floxacins was found to alter their solubility, bioavailability, and distribution in water and soil, which in turn is significant to the critical problem of resistance to this class of antibiotics.^41^

Antiviral drugs of the “gravir” family (Figure 6) feature a 1,3-dicarbonyl motif flanked by an additional oxygen donor and therefore present two adjacent metal-binding chelates. This essentially planar array of three oxygen donors interacts with the active site of HIV-1 integrases, in which two magnesium centers are coordinated by three carboxylate ligands of a characteristic aspartate-aspartate-glutamate (DDE) motif (Figure 6, inset).^54,55^ Antiviral baloxavir (Figure 6), which is approved to treat influenza, was developed based on these integrase inhibitors and shares their bis-bidentate structural motif. In this case, the three oxygen donors interact with two magnesium cations at the active site of an endonuclease that is critical for the viral cell cycle.^56^

Consistent with this mechanism of action, subtle changes in the geometry and/or Lewis basicity of the triad of donors were found to have a significant impact on inhibitory activity.^57^ When the metal coordination of gravir inhibitors was examined in solution, the metal complexes were found to have altered enzyme inhibitor activity as well as drug permeability in cultured mammalian cells and across rat intestinal mucosa.^58,59^ Indeed, oral coadministration of gravir drugs with metal-containing antacids and multivitamins may have a clinically significant impact on antiviral efficacy.^60^

The tetracyclines, anthracyclines and derivatives (Figures 7 and 8) commonly feature multiple vinylogous analogs of the 1,3-dicarbonyl motif or its aromatic variant 2-acyl phenol, which form 6-membered metal chelates similar to those of metal acetylacetonates. The ability of tetracycline antibiotics to coordinate transition metals has been documented extensively since the 1960s with 1:1 or 1:2 metal-to-ligand stoichiometry and different binding modes depending on the conditions in solution.^61,62^ A 1:1 magnesium complex is the active antibacterial species as the metal cation mediates the interactions between tetracycline and the sugar–phosphate backbone on the 30S subunit of the bacterial ribosome (Figure 7), thereby inhibiting protein synthesis.^63^ One of the mechanisms for resistance to tetracyclines involves enhanced transporter-mediated export of the magnesium complex; however, the coordination of tetracyclines to transition metals (e.g., Pd­(II), Pt­(II)) has shown promise to overcome this mechanism of bacterial resistance.^64^

The related glycoside drugs of the anthracycline class, such as doxorubicin and daunorubicin (Figure 8), are important, broad-spectrum chemotherapeutics currently included in the standard of care for multiple cancers. Anthracyclines have a complex mechanism of action, which includes well-recognized DNA intercalation and topoisomerase II inhibition as well as several other effects.^65,66^ The generation of reactive oxygen species (ROS) is a well-known aspect of anthracycline pharmacology, and it is attributed to the one-electron chemistry of the quinone/semiquinone redox couple and to the formation of redox-active iron complexes that engage in Fenton chemistry intracellularly. This iron-mediated oxidative stress is a key component of anthracycline-induced cardiotoxity, which is a major problem in the clinical use of this class of cancer chemotherapeutics.^67,68^ In particular, mitochondrial iron accumulation was implicated in the observed cardiotoxicity,^69^ and the clinical use of prochelator dexrazoxane, which hydrolyzes to produce a polydentate chelator, has cardioprotective effects.^70^ Furthermore, anthracyclines were found to impact iron signaling,^71^ and recent evidence points to a role of ferroptosis in doxorubicin-induced cardiomyopathy.^72^

A related bidentate motif with two oxygen donors was also identified on the naphthohydroquinone scaffold of the rifamycin drugs^73^ (Figure 8), which are first-line antibiotics for several infectious diseases including tuberculosis. Their primary mechanism of action entails the inhibition of prokaryotic RNA polymerases;^74,75^ however, the formation of metal complexes has been reported^76,77^ and associated with ROS generation and ensuing DNA damage in vitro.^78,79^ Metal-mediated ROS generation^80^ and its potential role in the development of antibiotic resistance^81^ were documented in cultured mycobacteria exposed to rifamycin drugs.

The 1,3-dicarbonyl, 2-acyl-phenol and related bidentate motifs capable of forming 6-membered chelates with two oxygen donors are also found in a variety of other small molecule drugs (Figure 9). Given the structural similarity to anthracycline drugs (Figure 8), it is not surprising that antineoplastic mitoxantrone has been implicated in iron coordination resulting in oxidative stress and cardiotoxicity.^82,83^ The metal coordination of mycophenolate immunosuppressants has been inferred spectroscopically;^84,85^ however, its relevance to gastrointestinal absorption of the drugs, particularly in the context of iron supplementation, is debated.^86,87^ Finally, although they feature a 1,3-keto–enol motif, anti-inflammatory drugs of the oxicam class have a well-documented coordination chemistry that typically involves bidentate chelates through the carbonyl donor and the nitrogen donor of the adjacent heterocycle (vide infra).^88,89^

**Figure 9:**
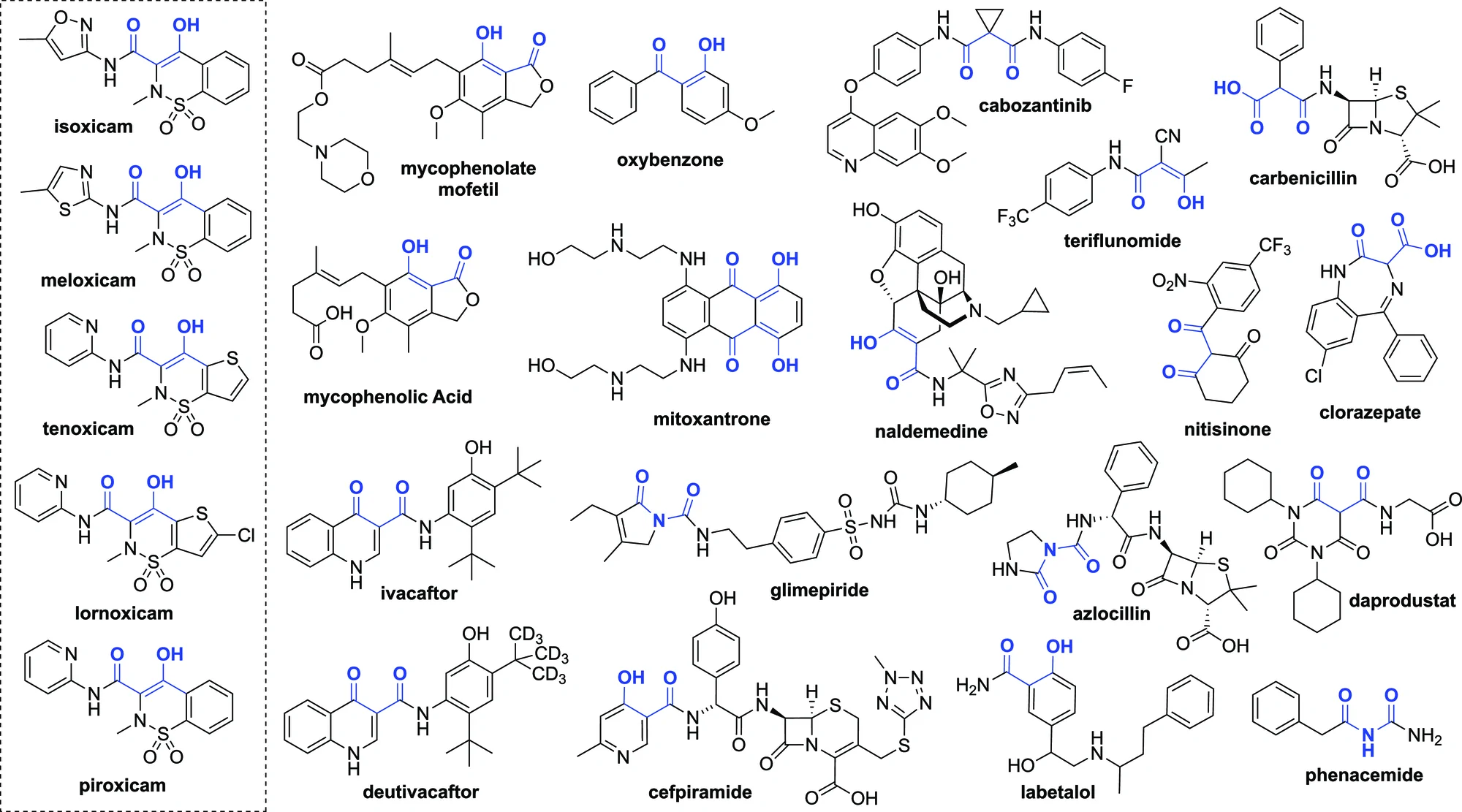
Various 1,3-dicarbonyl and related motifs in small molecule drugs.

The 2-hydroxybenzoate motif is a well-established chelating ligand found in salicylic acid and related drugs (Figure 10), including major NSAID aspirin, which is rapidly deacetylated in vivo. The metal affinities of salicylic acid have been known for decades,^90^ and the biological activities of many salicylate complexes have been reported.^91,92^ For instance, copper salicylate has been used in veterinary medicine,^93^ and related complexes have been investigated as anticancer agents.^94^ In addition, the bis-salicylate scaffold of olsalazine has been employed to build metal–organic frameworks for potential applications in drug delivery.^95^ Metal-binding effects (or interferences) in the mechanism of action of aspirin, however, remain to be determined, and their elucidation is likely complicated by the vast polypharmacology of this old drug, which is used in a wide range of dosages for its antipyretic, anti-inflammatory, analgesic, and antithrombotic effects. Furthermore, considerations of metal binding may also provide insights into epidemiological studies of aspirin as a chemoprotectant against several cancers.^96^

**Figure 10:**
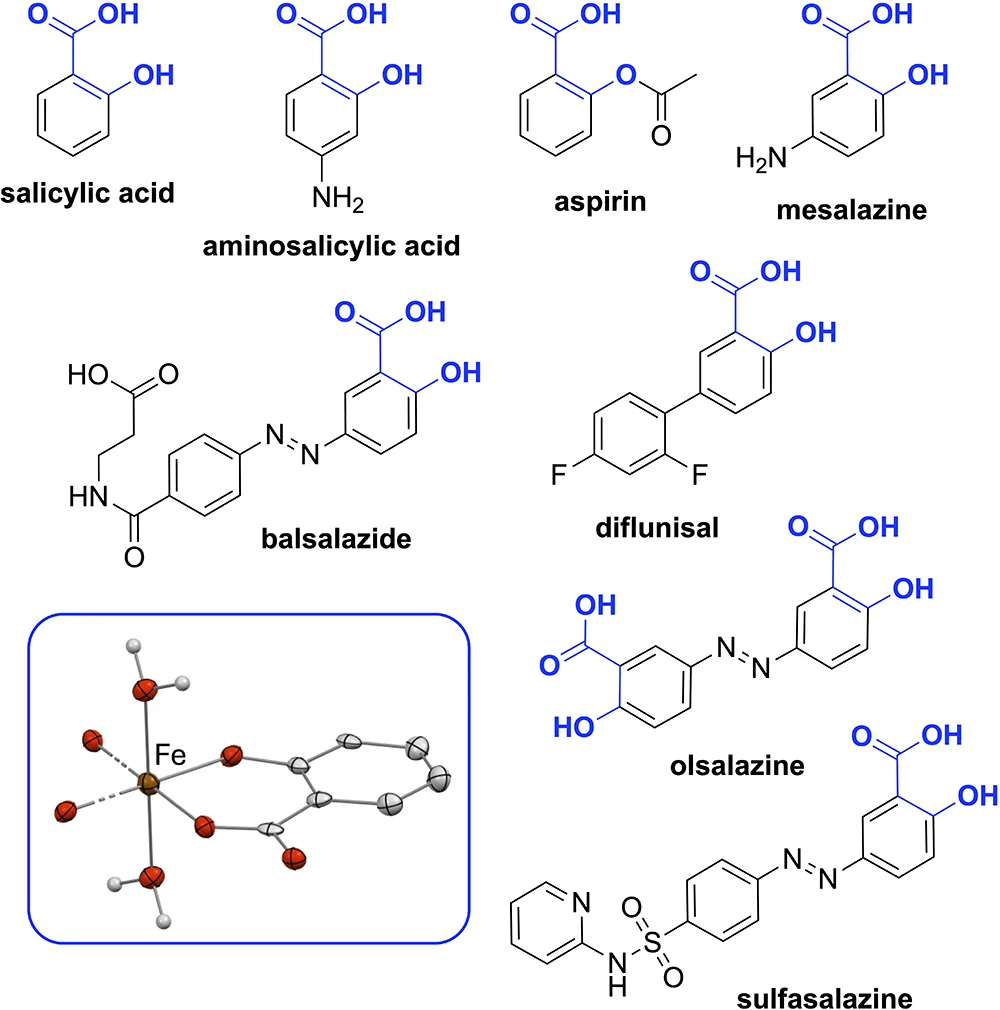
Salicylic acid motifs in small molecule drugs. Inset: crystal structure of a ferric complex featuring bidentate coordination of the salicylate motif.^300^ A symmetrically equivalent salicylate ligand, a hydrogen atom at half occupancy on the carboxylate donor, and all carbon-bound hydrogen atoms are not shown. CCDC: 1587792.

The *ortho*-dihydroxyphenyl motif of catechols (Figure 11) is well represented in FDA-approved drugs^97^ and its metal-binding properties are well established. For instance, the formation of multiple iron-catechol chelates by naturally occurring siderophores, such as enterobactin and petrobactin, is critical to iron acquisition by microorganisms.^98^ This metal-binding motif also appears in several neuromodulators of the catecholamine family, including dopamine and epinephrine, and related drugs, such as levodopa (Figure 11). Iron coordination and redox chemistry are part of the physiology of dopamine^99^ and epinephrine,^100,101^ with consequences on aging, cardiovascular health, and neurodegeneration. Consistently, the interactions of oral iron supplements with levodopa and methyldopa in the treatment of Parkinson’s disease (PD) have been known for decades.^102,103^ Notably, levodopa is often used in combination with catechol-containing entacapone (Figure 10), which also forms a stable ferric complex,^104^ and was recently found to alter the gut microbiome through iron sequestration.^105^ The iron-dependent effects of catechol-*O*-methyltransferase inhibitors entacapone and tolcapone (Figure 11) on the microbiome also impact the metabolism of coadministered levodopa,^106^ thus highlighting the complexity of metal-drug interactions with the gut microbiome. Apomorphine (Figure 11) is another catechol-containing drug for PD, and its antioxidant and neuroprotective properties have been linked at least in part to metal coordination.^107^

**Figure 11:**
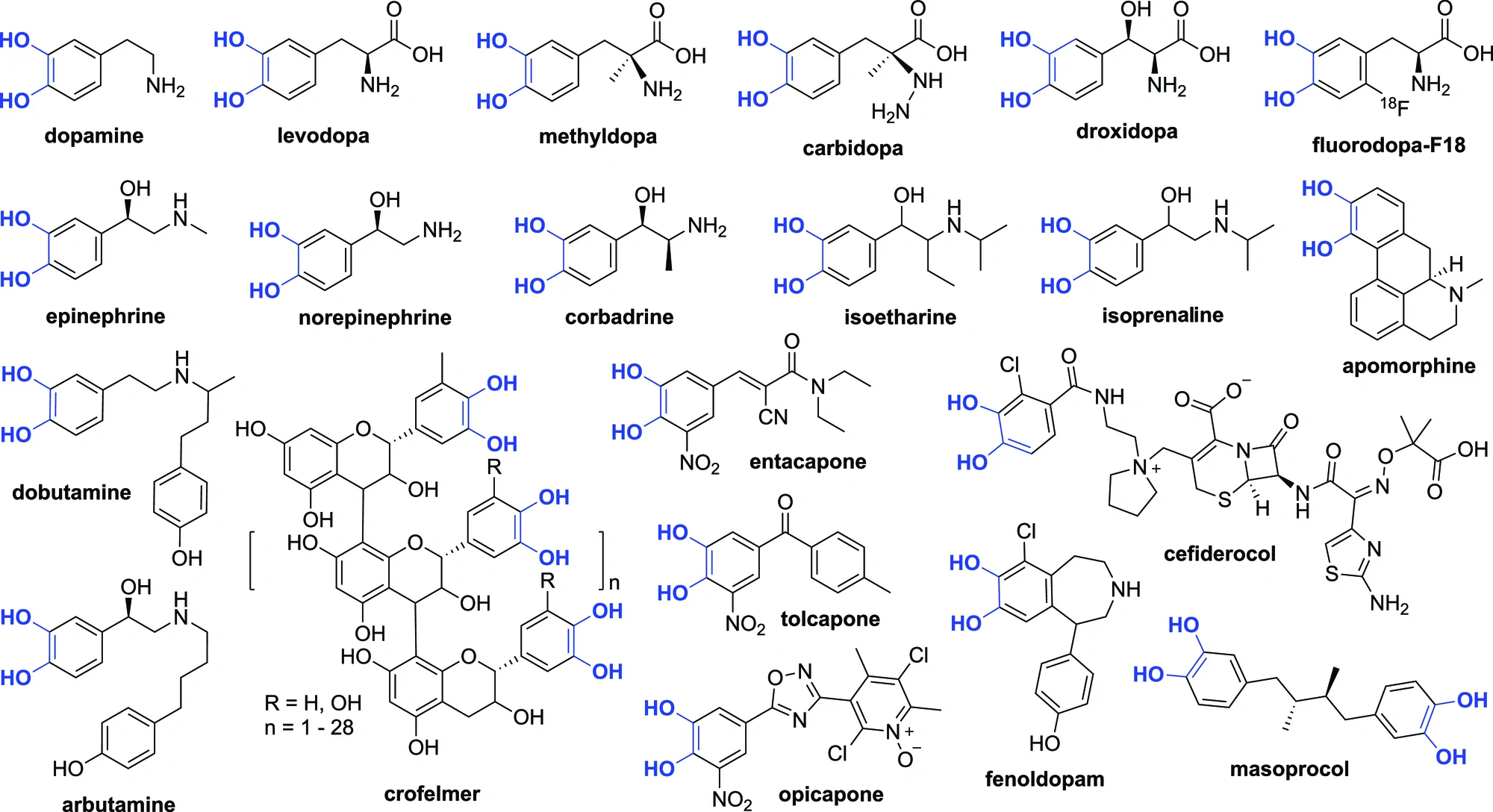
Catechol motifs in small molecule drugs.

The metal-binding properties of the catechol motifs are perfectly embodied by cefiderocol (Figure 11), a first-in-class antibiotic that combines a catechol-based siderophore moiety and a cephalosporin core.^108^ Upon binding extracellular iron, the catecholate complex acts as a Trojan horse for transporter-mediated uptake into the bacterial periplasmic space.^109^ Cefiderocol was FDA approved in 2019 for the treatment of complicated urinary tract infections, and several other indications are under consideration. Additional siderophore-drug approaches^110^ are likely to showcase the use of metal coordination (through catecholates, hydroxamates and other siderophore binding motifs) to facilitate antibiotic delivery to specific pathogenic microorganisms, particularly multidrug-resistant strains of Gram-negative bacteria.^111^

The bisphosphonate analogs of natural pyrophosphates form a unique class of bidentate metal-binding motifs featuring oxygen donors (Figure 12). Because of their affinity for hydroxyapatite and calcium, bisphosphonates are widely employed for the treatment of skeletal diseases, including osteoporosis, metastatic bone cancer, and heritable skeletal disorders in children.^112,113^ In addition, for the nitrogen-containing compounds in this class, such as zoledronate and risedronate (Figure 12), the interaction with magnesium ions is critical to the mechanism of action through the inhibition of farnesyl pyrophosphate synthase in osteoclasts.^114^ The ability of bisphosphonates to coordinate transition metals is also well documented, for instance in the case of copper^115^ and zinc.^116^ Although the clinical relevance of these interactions remains to be determined, several complexes have been investigated as potential agents against Chagas disease,^117^ and coordination polymers have been evaluated in the context of osteolytic metastases.^118^

**Figure 12:**
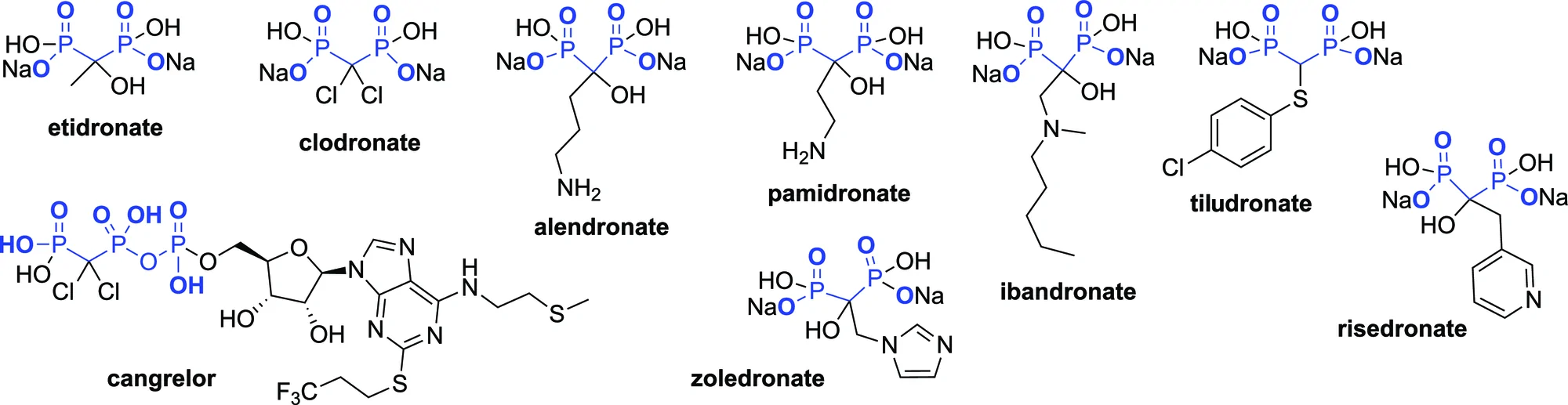
Bisphosphonate motifs in small molecule drugs.

### Bidentate Motifs Featuring at Least One Nitrogen Donor

Pyridine is the most commonly occurring nitrogen heterocycle in US FDA approved drugs^119,120^ and also an excellent ligand in coordination chemistry, therefore it is not surprising that many 2-substituted pyridines appeared in our survey of bidentate metal-binding motifs. Presented in Figure 13 are pyridine drugs with 2-aminopyrimidine or 2-aminoacyl groups that allow for 5-membered chelate rings upon coordination with (*N*,*N*) or (*N*,*O*) donor sets. In Figures 13–18, heterocycle rings have been color-coded so that the same heterocycle can be located easily through a variety of bidentate motifs and figures.

**Figure 13:**
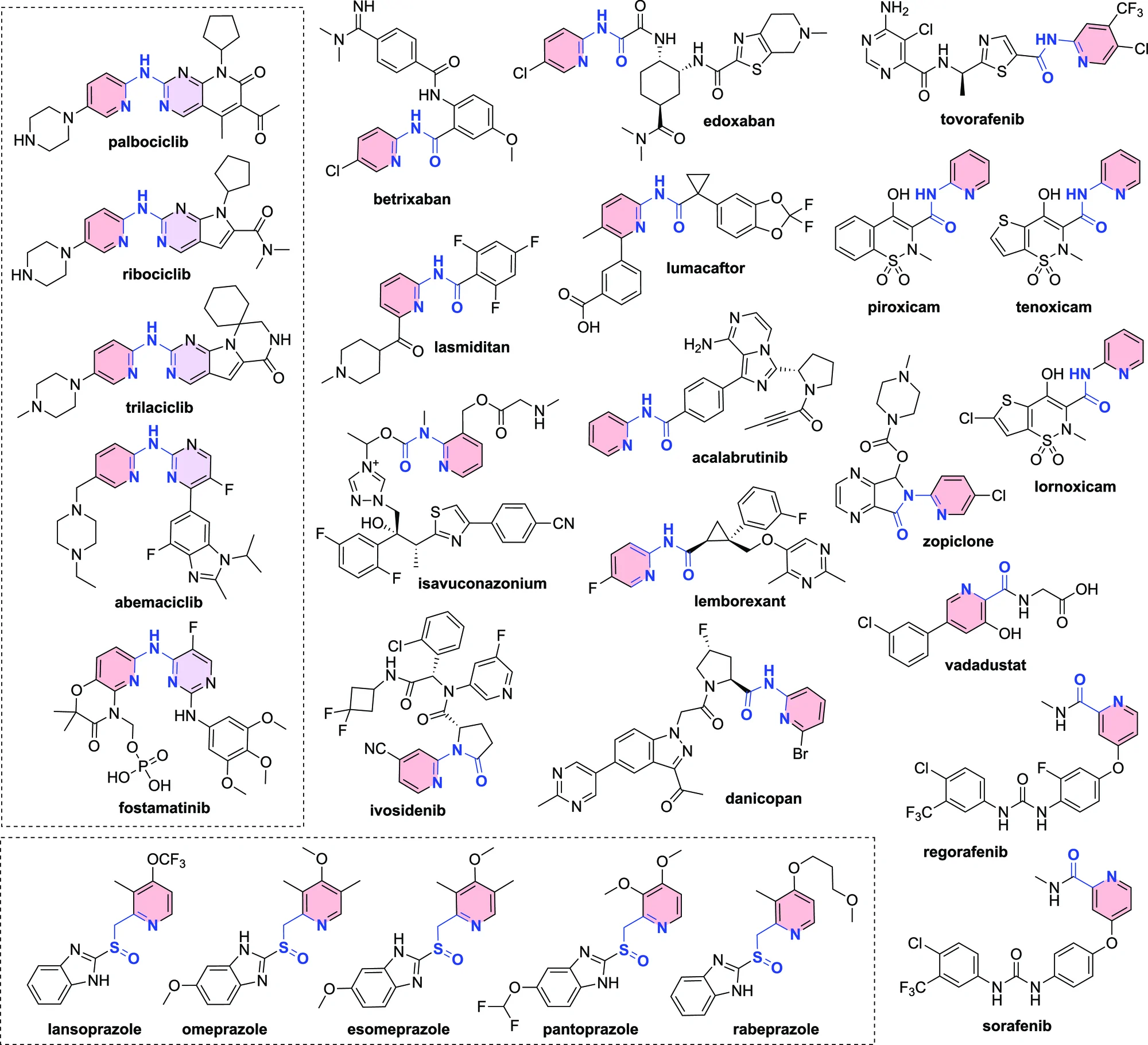
Pyridine-containing small molecule drugs with 2-amino-acyl, sulfoxide or 2-pyrimidine groups.

Kinase inhibitor and breast cancer drug palbociclib and related compounds (Figure 13) are structurally related to many compounds of the 2,2-dipyridylamine class of ligands for coordination chemistry.^121^ The primary mechanism of action of these inhibitors of cycline-dependent kinase-4 and −6 (i.e., CDK4/6) does not involve metal coordination and indeed entails binding to the hinge region of the target protein through multiple noncovalent interactions, including two critical hydrogen bonds to the 2-aminopyrimidine motif.^122^ The presence of metal ions, however, was found to affect the binding of ribociclib to plasma protein human α-1-acid glycoprotein,^123^ and it may generally affect secondary aspects of the complex pharmacology of this class of CDK inhibitors.^124,125^

The pyridine 2-acetamide motif, which coordinates several divalent transition metals,^126,127^ is also well represented in pyridine-containing drugs (Figure 13). Vadadustat (Figure 13) is approved for the treatment of anemia associated with chronic kidney disease and acts as an inhibitor of prolyl hydroxylase (PHD) by coordinating active-site iron through the pyridine nitrogen donor and the adjacent glycinamide oxygen.^128^ The potential for off-target iron coordination is recognized,^129^ and iron supplements are in the list of drug interactions for vadadustat. The pyridine-based binding motif of metalloprotein inhibitor vadadustat is also found in multikinase inhibitors sorafenib and regorafenib, which are employed for the treatment of several cancers. Some effects of these compounds on cellular iron levels have been noted in the context of ferroptosis studies; however, their efficacy as ferroptosis inducers is still being debated.^130,131^ Interestingly, ruthenium­(II) complexes of sorafenib are being investigated as prodrugs^132^ or epidermal growth factor receptor (EGFR) inhibitors.^133^

Omeprazole, rabeprazole, and related compounds (Figure 13) are an important class of proton pump inhibitors employed globally for the treatment of common disorders associated with gastroesophageal acid. Their scaffold features benzimidazole and sulfoxide donors in addition to the pyridine ring, therefore it is not surprising that metal binding is well documented in vitro.^134,135^ Interestingly, a recent chemoproteomic analysis indicated that these compounds form conjugates with cysteines in zinc finger proteins though an activation mediated by zinc coordination in the absence of acidic conditions.^136^ Such interactions with metalloproteins could therefore contribute to off-target effects for this important class of pharmaceuticals.

2,2′-Bipyridine (often simply indicated as bpy in chemical formulas) is the parent of a major class of bidentate ligands in coordination chemistry^137,138^ with far-reaching applications from supramolecular chemistry to photocatalysis. For instance, bipyridine ligands are found in the ruthenium-based photosentitizer TLD1433, which is currently in advanced clinical trials for bladder cancer treatment.^139^ Interestingly, the simple 5,5′-dimethyl-2,2′-bipyridine (abametapir, Figure 14) is approved for the treatment of head lice^140^ with a mode of action relying on metal binding and metalloproteinase inhibition.^141^ Several other approved drugs feature a pyridine connected at the 2- position to another heterocycle potentially allowing for a five-membered metallacycle (Figure 14).

**Figure 14:**
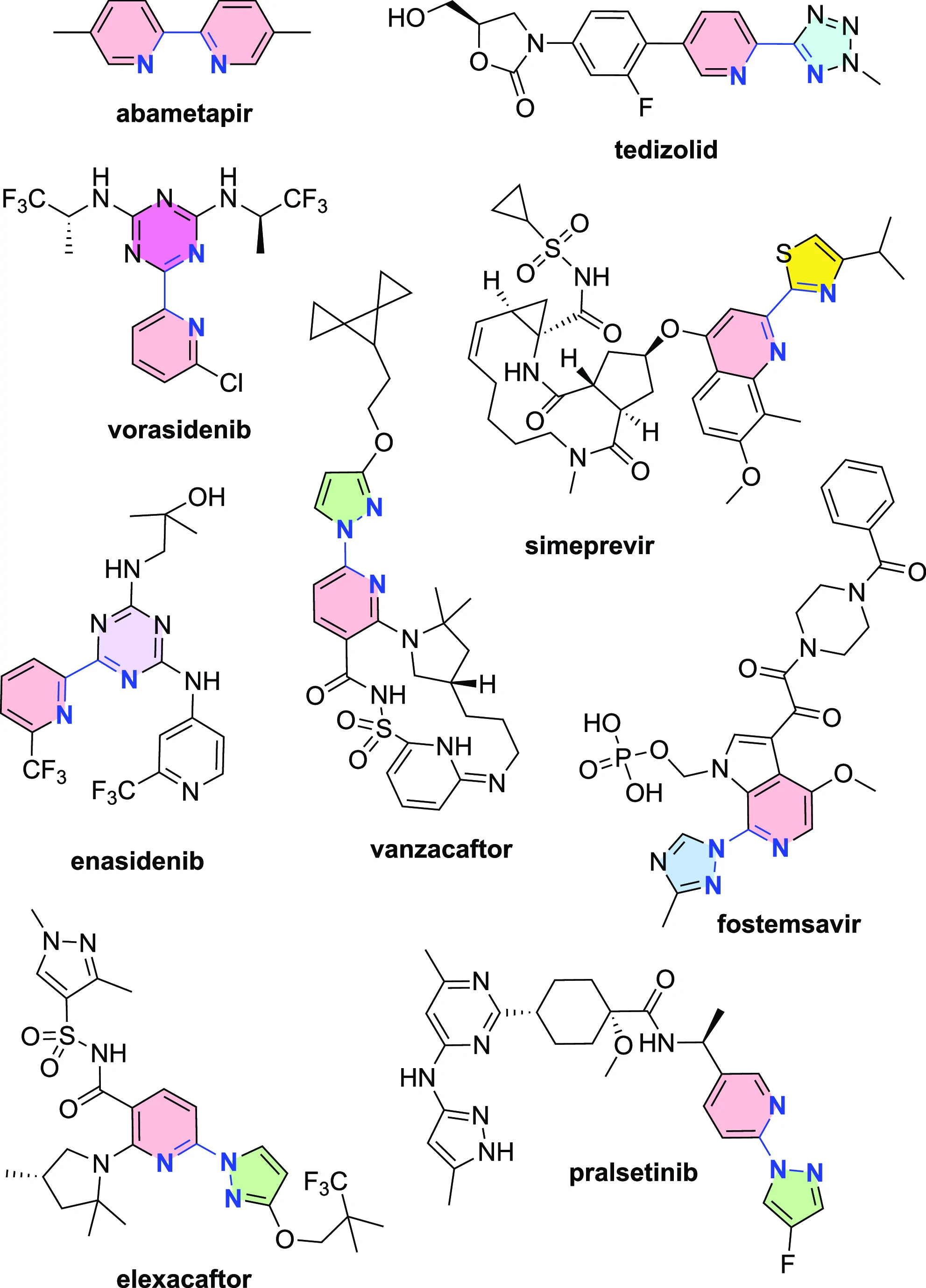
2-Pyridyl bidentate motifs connected to various heterocycles in small molecule drugs.

A 1,3-diazine (i.e., pyrimidine) ring is part of the metal-binding unit within the elaborate structure of bleomycin (Figure 15), a glycopeptide antibiotic that is employed in combination chemotherapies for multiple cancer indications.^142^ In this case, a bidentate motif is obtained through the adjacent amidic nitrogen. The nearby imidazole and primary amide contribute to the coordination of endogenous Fe­(II) or Cu­(I),^143,144^ thereby forming a catalytic center of ROS production resulting in DNA damage.^145^ Indeed, bleomycins are examples of highly sophisticated drugs and natural products featuring structures that not only optimize cellular uptake and DNA intercalation but also recruit redox-active metals to generate oxidative stress catalytically.

**Figure 15:**
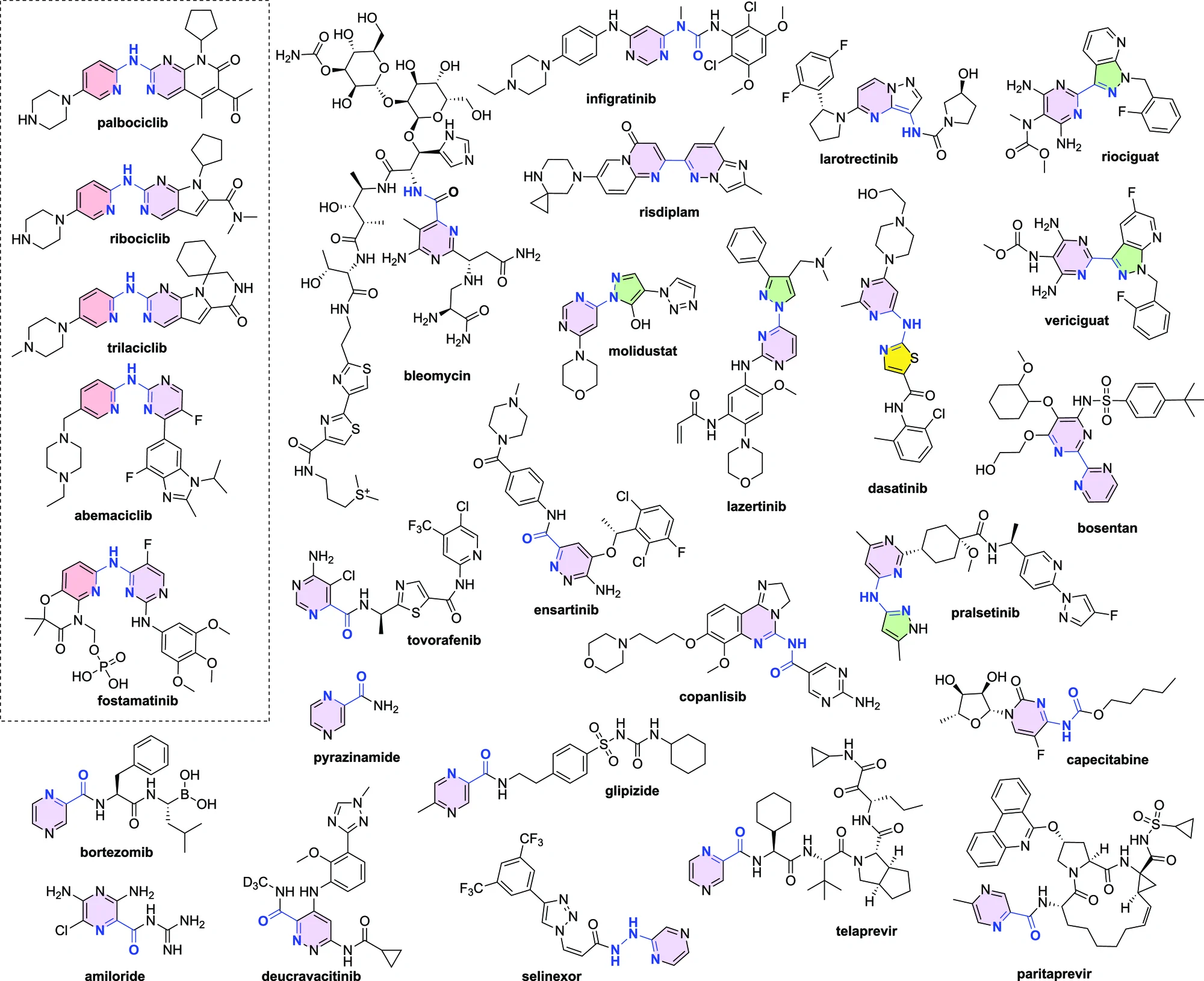
Bidentate motifs containing pyridazine (1,2-diazine), pyrimidine (1,3-diazine), and pyrazine (1,4-diazine) heterocycles.

As in the metal-binding unit of bleomicyin, an amide group is found on the diazine ring of several kinase inhibitors such as tovorafenib and ensartinib (Figure 15). In addition, the simple pyrazinamide molecule is a known ligand in coordination chemistry^146^ and a frontline antituberculosis drug.^147^ Interestingly, the activities of pyrazinamide and its metabolite pyrazinoic acid against *M. tuberculosis* are enhanced by iron supplementation in vitro.^148^ An analogous bidentate motif is present in anticancer drug bortezomib, which was found to be impacted by iron levels in multiple myeloma cells and associated macrophages.^149–151^

A 2-amino-pyridyl group is connected to pyrimidine rings on several structures previously mentioned for pyridine-containing drugs (Figure 13). A related amino-thiazolyl pyrimidine motif appears in potent tyrosine kinase inhibitor dasatinib. This bidentate motif forms a Cu­(II) complex, which was isolated and found to have antiproliferative activity in several cancer cell lines.^152^

The pyrimidine heterocycle is critical for the mechanism of action of FDA-approved veterinary drug molidustat (Figure 15), a PHD inhibitor that coordinates iron at the enzyme active site through the nitrogen donors of the pyrimidine and adjacent pyrazolone.^153^ Interestingly, an analogous motif is found in tyrosine kinase inhibitor lazertininb. Direct connections of a pyrimidine to another heterocycle forming potential bidentate units are also present in other examples in this figure, including riociguat, vericiguat, risdiplam, and bosentan.

Thiazoles are highly represented in FDA approved drugs^106,107^ and also in our survey, where they appear frequently flanked by an oxime in third-generation β-lactam antibiotics (Figure 16). Although the coordination chemistry of thiazoles is well documented,^154^ these antibiotics offer a variety of other potentially metal-binding groups. In general, the possibility of metal interactions is often acknowledged in their clinical guidelines, which typically recommend against coadministration with iron supplements.^155^ Notably, two of these antibiotics feature a thiadiazole heterocycle, which is also found in the structure of acetazolamide (Figure 16), a carbonic anhydrase inhibitor that interacts with zinc at the enzyme active site through the sulfonamide group.^156^

**Figure 16:**
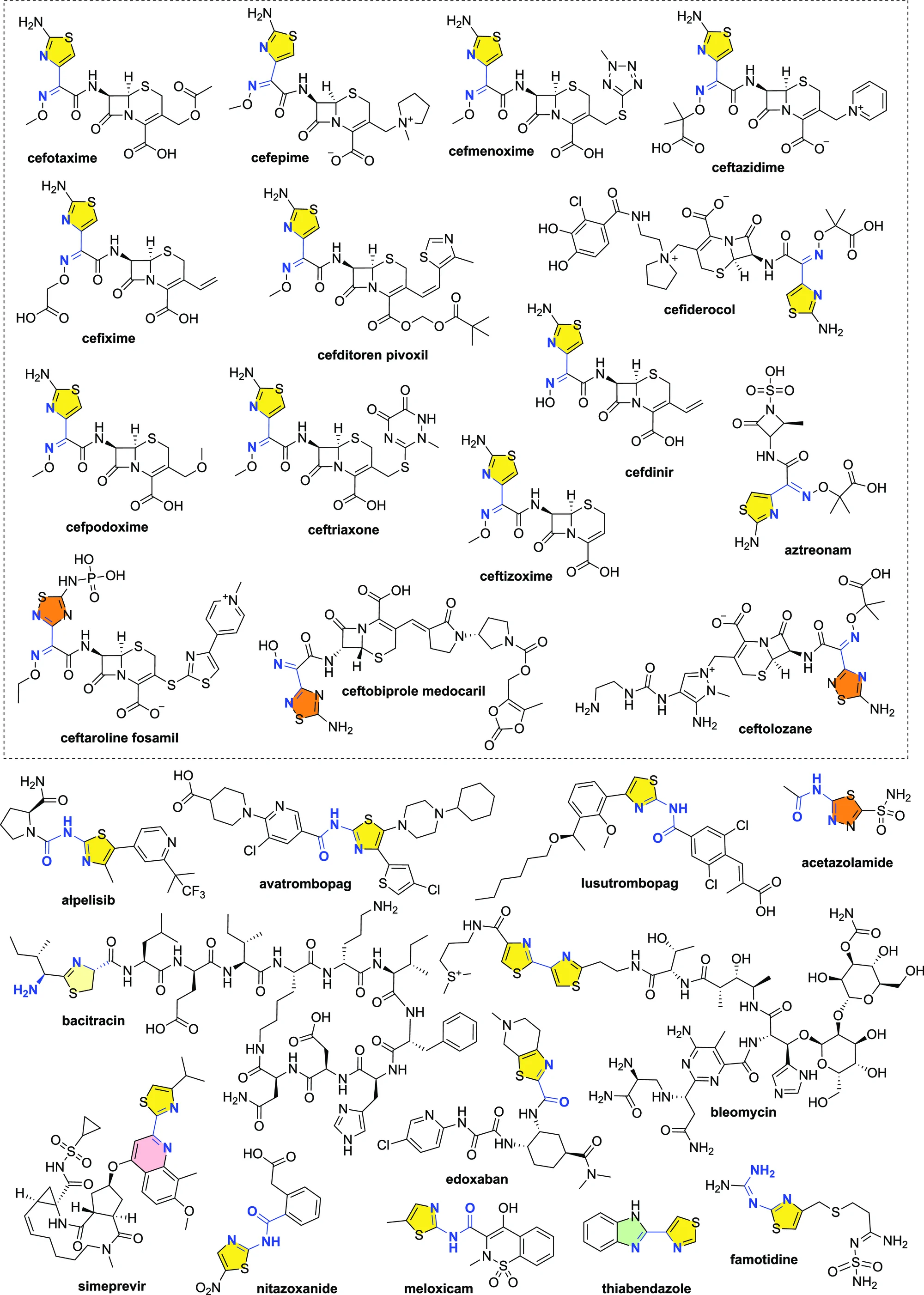
Thiazole and thiadiazole bidentate motifs in small molecule drugs.

Thiazole heterocycles are also found in the DNA-intercalating region of bleomycin and in the recognized metal-binding motif of anti-inflammatory drug meloxicam (vide supra). Antiparasitic and fungicidal compound thiabendazole (Figure 16) is a known metal-binding species,^157–159^ although the clinical relevance of metal coordination is not established. In a unique motif in this survey, the thiazoline ring is a known metal-binding group^160^ within the complex structure of cyclic peptide bacitracin (Figure 16), a widely used antibiotic. Interestingly, bacitracin is typically sold in a zinc-containing formulation, and its antimicrobial activity is attributed to the formation of a ternary complex with zinc and lipid-pyrophosphates, whereby the metal center coordinates the thiazoline nitrogen and the adjacent primary amine.^161^

Within the group of imidazole, pyrazole, and isoxazole motifs (Figure 17), we find several previously discussed structures, including thiabendazole, the “prazole” family, and isoxicam (vide supra). Among the new motifs in this group, antiviral acyclovir was isolated in numerous metal complexes, in which it primarily coordinates only through the nitrogen donor.^162^ Bidentate acyclovir coordination, however, was also reported through the adjacent carbonyl oxygen,^163,164^ and some biological activity of the complexes has been noted.^165,166^ In the case of cancer chemotherapeutic temozolomide, a biologically active copper complex of drug metabolite 5-(3-methyltriazen-1-yl)­imidazole-4-carboxamide was proposed, but the coordination mode was not characterized fully.^167^ Bidentate coordination was documented for antiemetic granisetron,^168^ although its biological relevance remains to be determined.

**Figure 17:**
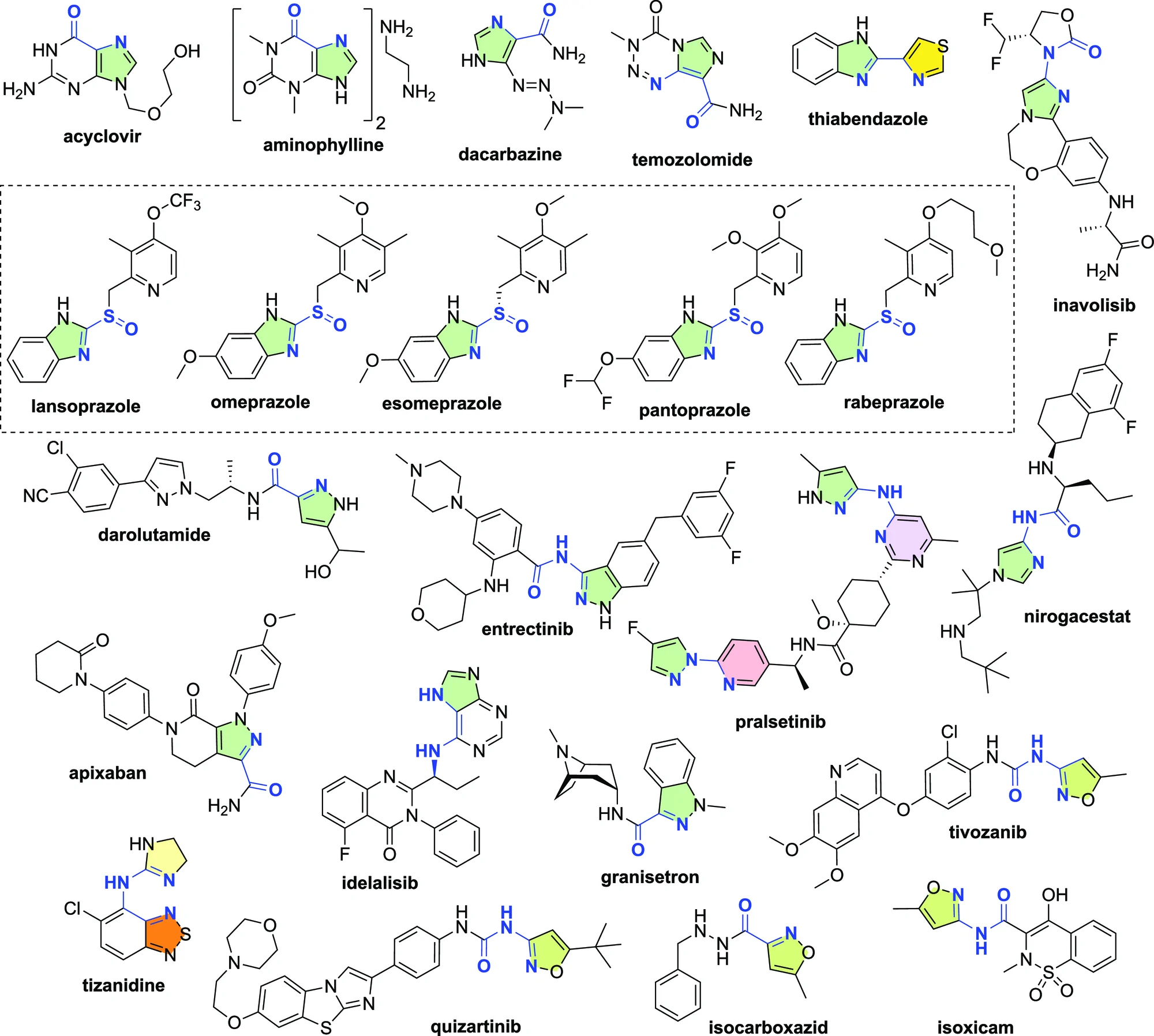
Imidazole, pyrazole, and isoxazole containing motifs in small molecule drugs.

Triazole rings (i.e., 1,2,3-triazole and 1,2,4-triazole, Figure 18) are common in medicinal chemistry and their metal-binging ability is well recognized.^169^ Within this group in our survey, deferasirox chelates iron as a high-affinity tridentate ligand and is widely used for the treatment of systemic iron overload associated with hemochromatosis and transfusion-dependent thalassemia.^170^ Antifungals of the azole family, such as fluconazole and voriconazole (Figure 18), inhibit fungal cytochrome P450 enzyme lanosterol 14α-demethylase (CYP51) through axial coordination of a single triazole nitrogen to heme iron at the active site.^171^ Triazole nitrogen donors in these fungistatic drugs serve as monodentate ligands in many transition metal complexes that are being investigated as bioactive compounds.^172,173^ Tridentate fluconazole coordination has been observed in a binuclear copper complex (Figure 18);^174^ however, copper was found to potentiate fluconazole antifungal activity even in the absence of complex formation.^175^ More generally, a critical interplay between azole susceptibility and metal homeostasis is increasingly recognized.^176–178^ Copper coordination was also reported for antiviral ribavirin (Figure 18);^179^ however, the biomedical relevance of metal binding is not well-defined for this and other motifs in this group, which also includes a few tetrazoles.^180^

**Figure 18:**
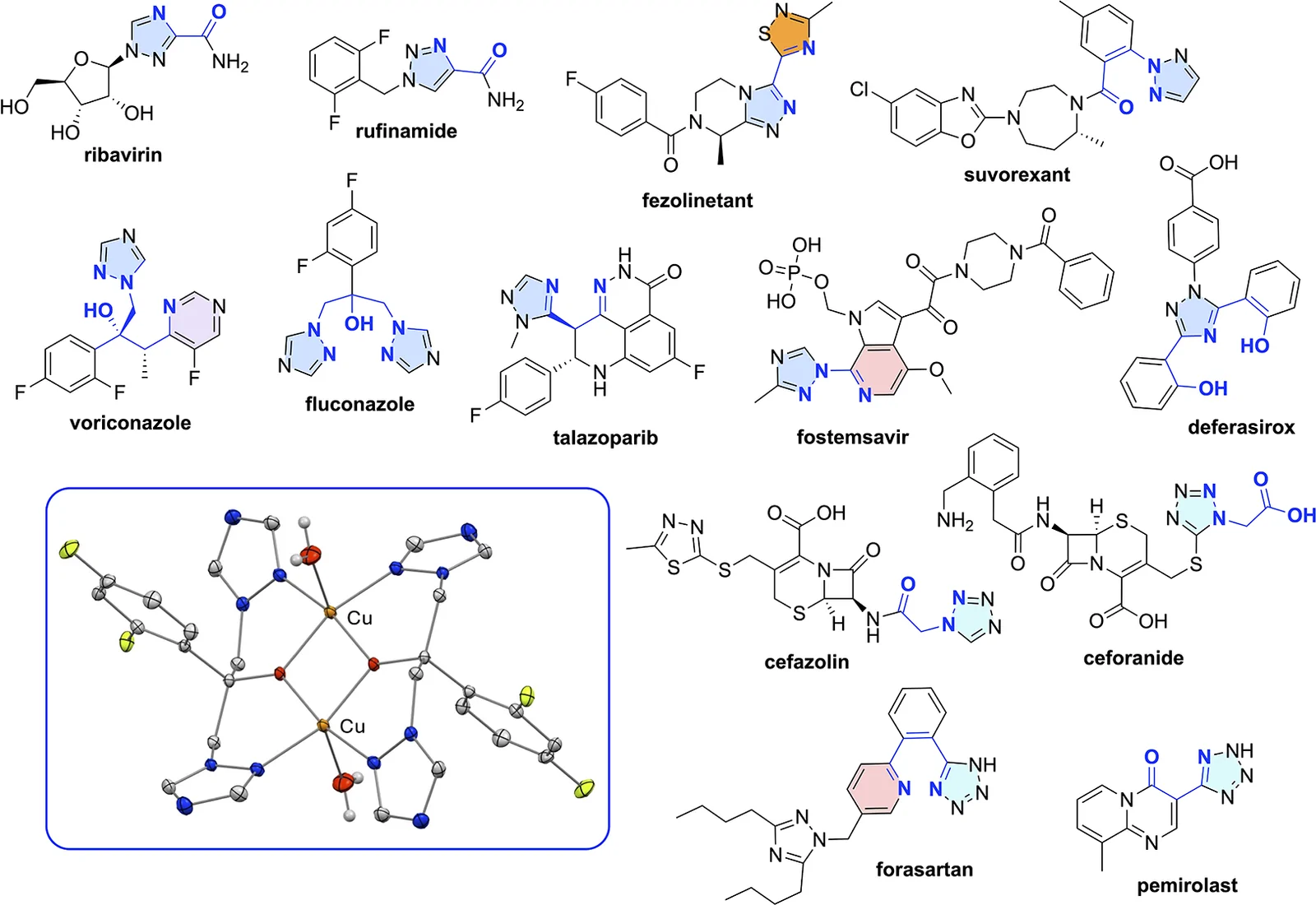
Triazole- and tetrazole-containing motifs in small molecule drugs. Inset: crystal structure of a binuclear Cu­(II) complex featuring multidentate coordination of fluconazole with two bridging oxygen donors and two aqua ligands.^174^ Two nitrate counterions and all carbon-bounds hydrogen atoms are not shown. CCDC: 826112.

The biguanide family of drugs (Figure 19) present a bidentate metal-binding unit through two adjacent imine groups, which form a 6-membered chelate ring upon metal coordination. Within this group, metformin is an essential drug employed worldwide for the management of type-2 diabetes, and many of its metal complexes have been isolated and characterized (Figure 19).^181,182^ Although metformin has been used for decades, its mechanism of action is not fully understood. Recently, the binding of endogenous copper was found to be critical for the antihyperglycemic properties of metformin through the activation of AMP-activated protein kinase (AMPK) signaling and S6 protein phosphorylation.^183,184^ Interestingly, the antimalarial properties of several biguanide drugs were also connected to interactions with endogenous metals (i.e., copper, zinc, iron), particularly to the ability of biguanide–metal complexes to inhibit cysteine proteases in the *Plasmodium* parasites.^185^ The similar acylguanidine moiety of antihypertensive drugs guanfacine and amiloride (Figure 19) is also a metal-binding motif,^186,187^ although the pharmacological relevance of metal interactions is not well-studied.

**Figure 19:**
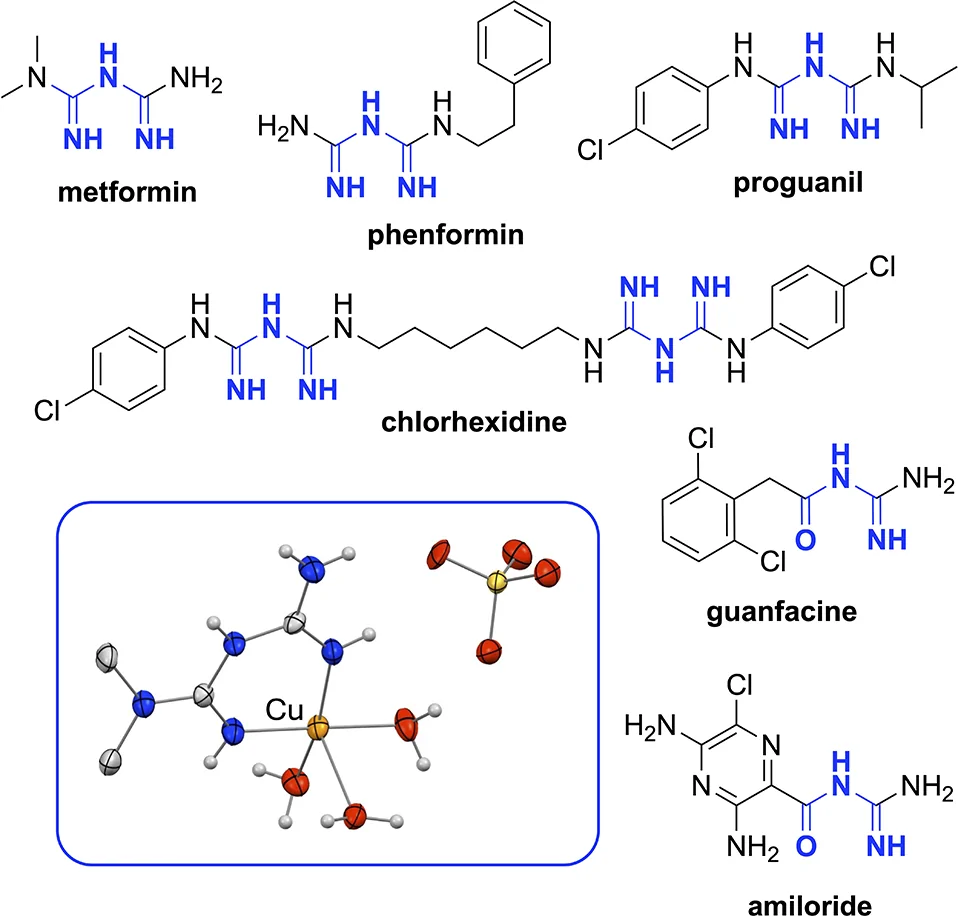
Biguanides and acylguanidine motifs in small molecule drugs. Inset: crystal structure of a Cu­(II) complex featuring a bidentate metformin ligand, three aqua ligands, and a sulfate counterion.^183^ Carbon-bound hydrogen atoms are not shown. CCDC: 2009030.

A collection of miscellaneous motifs (Figure 20) that do not fit in our previous groups contains several compounds with established metal coordination as well as drugs that have not been associated with metal binding.

**Figure 20:**
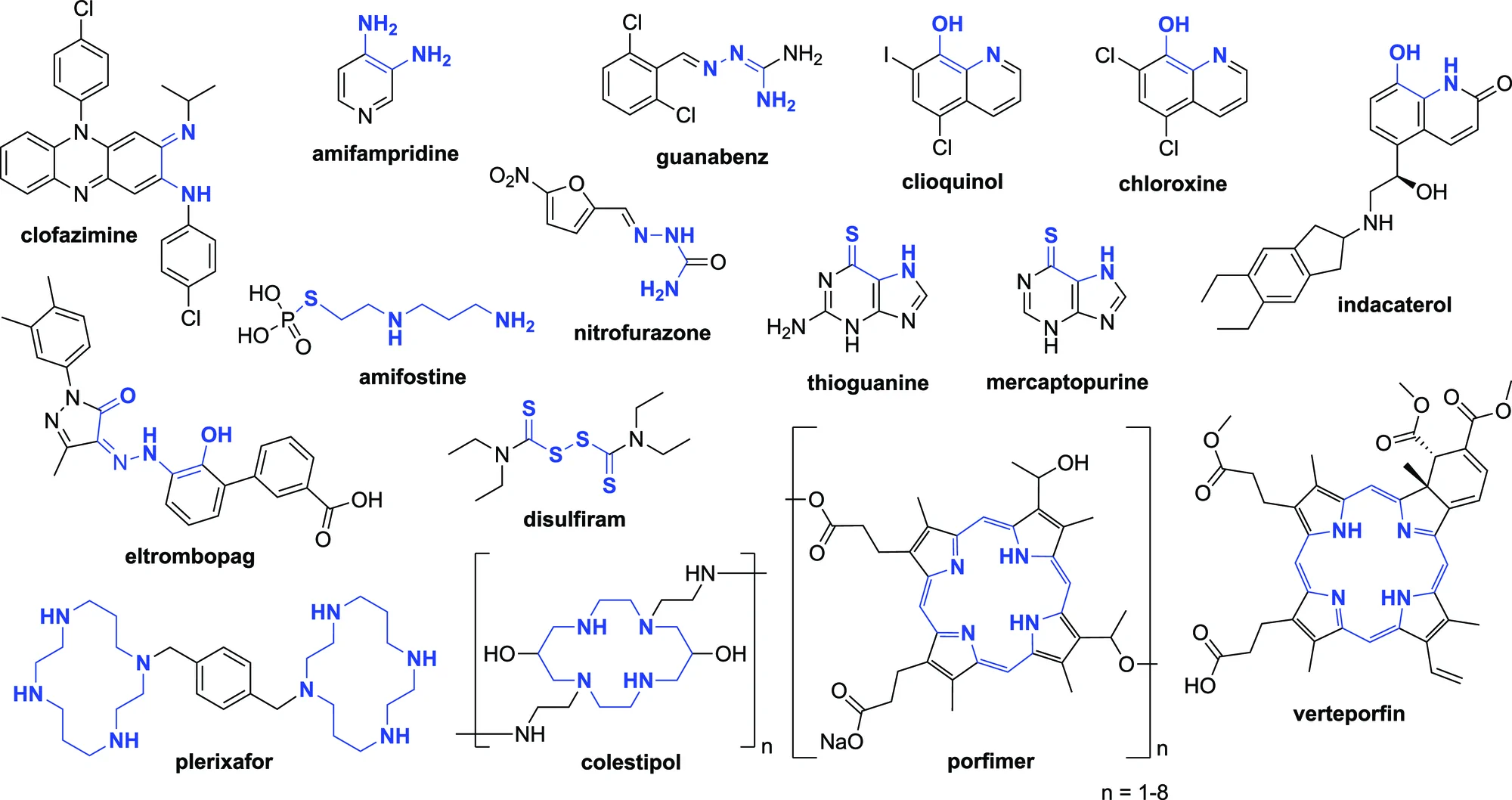
Miscellaneous bidentate and multidentate motifs in small molecule drugs.

Plerixaflor (aka AMD3100, Figure 20) is an antagonist of chemokine receptor CXCR4, which is relevant to multiple diseases ranging from AIDS to cancer to rheumatoid arthritis. Its cyclam rings coordinate several transition metals (e.g., Ni­(II), Cu­(II), Zn­(II)), and the metal complexes of this compound and related analogs were found to have enhanced affinity for CXCR4.^188,189^ A related azamacrocycle is found in colestipol (Figure 20); however, this anion exchange resin remains confined to the acidic environment of the intestine, where it is employed to sequester bile acids^190^ and is unlikely to coordinate metal ions. Tetraaza-macrocyclic structures are also characteristic of tetrapyrroles porfimer and verteporfin (Figure 20), two photosensitizers approved for photodynamic therapy in cancer and eye diseases. Although these compounds are currently employed as free bases, palladium tetrapyrrole padeliporfin was recently approved by the European Medicines Agency (EMA).^191^

Within the group of miscellaneous motifs, we find a few sulfur-containing units, which are present in several chelators (Figure 3) but not generally common throughout our survey. Purine antimetabolites mercaptopurine and thioguanine (Figure 20), which are employed broadly to treat certain leukemias and autoimmune diseases, coordinate several transition metals.^192^ Some of the complexes have shown biological activity in vitro but have not undergone clinical development.^193,194^

A sulfur-containing structure is also featured in disulfiram, an inhibitor of alcohol dehydrogenase used in the management of alcohol abuse disorder. The pharmacology of this drug is rather complex and multiple other indications are being pursued for applications ranging from parasitic and viral infections to cancer.^195^ The ability of disulfiram metabolite diethyl dithiocarbamate to coordinate copper^196^ is a well-documented component of its anticancer activity^197,198^ and forms the basis for several current approaches featuring dithiocarbamates and/or their metal complexes.^199–202^

Finally, a sulfur donor is present in radiation protection agent and cancer chemotherapy adjuvant amifostine, a prodrug that is activated through dephosphorylation by alkaline phosphatase to release 2-(3-aminopropylamino)­ethanethiol.^203^ The mechanism of action of amifostine is not fully understood; however, metal binding is often viewed as a contributing factor, including through the inhibition of matrix metalloproteinases.^204^

Among the remaining compounds with nitrogen and oxygen donors, eltrombopag is an agonist of the thrombopoietin receptor approved to treat thrombocytopenia, and its clinically relevant ability to bind iron has been recently documented.^205,206^ A role for iron interactions is also emerging within the complex mechanism of action of clofamizine,^207^ an antileprosy drug that is increasingly used in the treatment of tuberculosis.

The 8-hydroxyquinoline motif is a well-established chelating ligand, and its binding of endogenous transition metals is critical for the therapeutic action of clioquinol and chloroxine.^208,209^ These antifungal and antibacterial drugs are primarily employed as topical preparations; however, these and many related derivatives are under investigation for their ability to modulate metal homeostasis in multiple diseases, particularly in the context of neurodegeneration.^210,211^ Interestingly, the bronchodilator indacaterol appears structurally related to the 8-hydroxyquinoline drugs, but its metal binding properties are not well established.

## Conclusion

We surveyed the structures of the US FDA-approved small-molecule drugs to identify bidentate motifs that could in principle bind metal cations to form 5- and 6-membered chelate rings through the coordination of oxygen, nitrogen, and sulfur donors. This analysis highlights the large number of drugs with known metal-binding groups (e.g., hydroxamates, catechols, salicylates, and 1,3-dicarbonyls) and recognized metal interactions in clinical settings. Among numerous motifs featuring nitrogen heterocycles, pyridine and pyrimidine rings are known metal-binding groups in prolyl hydroxylase inhibitors and bleomycins, respectively. We also identified numerous motifs featuring nitrogen heterocycles for which the coordination of endogenous metals has been postulated and is under investigation. Examples include kinase inhibitors, proton pump inhibitors, broad-spectrum antibiotics, and antifungal agents. In a highly heterogeneous group of miscellaneous motifs, metal interactions are documented in several commonly used drugs, including metformin, clioquinol, and chemotherapy adjuvant amifostine. For some of the compounds selected by our survey, however, metal coordination may be clinically irrelevant even if metal complexes were synthesized and isolated in organic solvents. In spite of the necessary caveats, the collection of identified compounds showcases a remarkable structural diversity of bidentate motifs in FDA approved drugs. It is our hope that this survey will serve as a useful source of metal-binding motifs in drug architectures and as a starter of conversations on metals in drug discovery through molecular considerations, instructive examples, and unexplored possibilities.

## Abbreviations and Acronyms

AMPK: AMP-activated protein kinase
BCE: before common era
CDK4/6: cycline-dependent kinase-4 and -6
DDE: aspartate-aspartate-glutamate
DNA: deoxyribonucleic acid
EDTA: ethylenediaminetetraacetic acid
EGFR: epidermal growth factor
EMA: European Medicines Agency
US FDA: United States Food and Drug Administration
HDAC: histone deacetylases
INN: International Nonproprietary Name
MRI: magnetic resonance imaging
NSAID: nonsteroidal anti-inflammatory drug
PAINS: pan-assay interference compounds
PD: Parkinson’s disease
PET: positron emission tomography
PHD: prolyl hydroxylase
RNA: ribonucleic acid
ROS: reactive oxygen species.
